# Synthesis and Antidiabetic Evaluation of Novel 2,4-Thiazolidinedione Derivatives Targeting Key Carbohydrate-Digesting Enzymes

**DOI:** 10.3390/molecules31183160

**Published:** 2026-09-08

**Authors:** Mahendra Gowdru Srinivasa, Shreya Kanchan, Darshan S, Karthik G. Pujar, Gurubasavaraj V. Pujar, Prashant Nayak

**Affiliations:** 1 Nitte (Deemed to Be University), NGSM Institute of Pharmaceutical Sciences (NGSMIPS), Department of Pharmaceutical Chemistry, Mangalore 575018, India; mahendra@nitte.edu.in (M.G.S.); shreyakanchan@nitte.edu.in (S.K.); darshan.21upl015@student.nitte.edu.in (D.S.); 2 Department of Pharmaceutical Chemistry, JSS College of Pharmacy, JSS Academy of Higher Education and Research, Mysuru 570015, India; karthikgv@gmail.com (K.G.P.); gvpujar@jss.edu.in (G.V.P.); 3 Nitte (Deemed to Be University), NGSM Institute of Pharmaceutical Sciences (NGSMIPS), Department of Pharmaceutics, Mangalore 575018, India

**Keywords:** ADME studies, antidiabetic activity, molecular docking, thiazolidinedione derivatives, α-glucosidase inhibition

## Abstract

Diabetes mellitus is a long-term metabolic disease associated with elevated glucose levels in blood and still constitutes one of the major public health issues worldwide. Inhibition of carbohydrate-digesting enzymes like α-amylase and α-glucosidase has been found to be effective in controlling postprandial hyperglycemia. The current study focused on designing, synthesis, characterization, and evaluation of novel 2,4-thiazolidinedione derivatives (**D1**–**D5**) as potent antidiabetic drugs utilizing combined in silico, in vitro, and in vivo techniques. Results from drug-likeness and ADME analyses indicated that all synthesized derivatives met Lipinski’s rule of five and had desirable pharmacokinetics properties along with reduced toxicity. Molecular docking against maltase-glucoamylase (human; PDB ID: 3TOP) protein showed good binding affinities of both **D1** and **D5** derivatives (−7.74 and −7.40 kcal/mol respectively) due to stable interactions with catalytic residues of enzymes. Inhibition of enzymes in vitro showed that **D1** and **D5** had the highest inhibitory activities of all synthesized derivatives, with IC_50_ of 33.86 ± 2.1 and 37.55 ± 1.7 μM against α-amylase and 29.81 ± 3.2 and 32.43 ± 1.2 μM against α-glucosidase, respectively. Cytocompatibility tests on L6 myoblast cells proved that the lead compounds were well tolerated. In addition, studies in a model of *Drosophila melanogaster* induced by a high-sugar diet revealed a significant decrease in the level of glucose concentration depending on the dose, especially for **D1** and **D5**, indicating their antihyperglycemic activity in vivo. Thus, these data confirm that **D1** and **D5** can be regarded as promising lead compounds for the development of new antidiabetics acting via inhibition of carbohydrate-metabolizing enzymes.

## 1. Introduction

Diabetes mellitus (DM) is widely acknowledged as a complicated illness that is becoming more widespread and has reached epidemic proportions. All age groups have been equally affected. In 2015, 415 million people worldwide were estimated to have diabetes by the International Diabetes Federation; by 2040, that figure is predicted to increase to over 640 million [1,2]. DM is a chronic condition marked by persistently high blood glucose levels, triggered by insufficient secretion of insulin, impaired insulin sensitivity, or a combination of the two. Recognized as a critical international health crisis and a primary driver of death, it currently affects an estimated 422 million people globally [3].

The burden of this disease is especially significant in low- and middle-income nations, where its prevalence continues to rise. Chronic hyperglycemia brought on by diabetes causes proteins to become glycated, which can lead to issues (damage) with the kidneys, arteries, nerves, and eyes [4]. The various types of this disease include type I diabetes mellitus (T1DM), type II diabetes mellitus (T2DM), gestational diabetes mellitus (GDM) and other forms. Type 1 diabetes is caused by genetic and/or epigenetic susceptibility factors. Type 2 diabetes is caused by a variety of triggering factors, especially those associated with the foods we eat on a daily basis [5,6,7,8]. Type 2 diabetes represents the most prevailing form of the condition, comprising more than 90% of total diagnoses. This specific variation typically develops due to the body’s incapacity to produce adequate amounts of insulin or its failure to respond effectively to the insulin that is present. Poor insulin production, the body’s inability to use insulin, or a combination of the two are the main causes of diabetes (insulin is a hormone that regulates the metabolism of proteins, fats, and carbohydrates) [9]. Cardiovascular, hypertensive, renal, and neurological problems are among the many clinical manifestations of this usually chronic syndrome. Cells in this condition are unable to absorb blood glucose because either the cells do not react to the secreted insulin, or the pancreas does not secrete enough of it. Its symptoms include hunger, exhaustion, excessive dehydration, and frequent urination. It can be cured by changing eating patterns, eating a balanced diet, taking oral medicine, and in some situations, getting insulin injections [10]. Diabetes management, especially type 2 diabetes mellitus, can be achieved through delaying glucose absorption by inhibiting enzymes responsible for the breakdown of carbohydrates in the digestive tract, for instance, α-glucosidase. The mechanism through which the effect is attained is by preventing the conversion of complex carbs to simple sugars within the body [11]. Studies have shown that managing high post-meal blood sugar is more impactful in lowering the burden of heart diseases than managing fasting blood glucose levels alone. The reason behind this is that there is an association between the rise in blood glucose after eating and the development of diabetes complications [12]. Post-meal high blood sugar can be reduced by blocking the enzymes that hydrolyze carbohydrates. Products with a small molecular weight like maltose, glucose, and maltotriose are produced when the starch’s α-1,4-glycosidic linkages are broken down randomly in locations by a metalloenzyme called α-amylase. α-amylases are generated in the pancreas and salivary glands. α-glucosidase catalyzes the breakdown of oligosaccharides, disaccharides, or aryl or alkyl-glucosides into smaller sugar particles, such as glucose [13]. It is one of the brush borders in the small intestine [6,7]. Acarbose, Miglitol, and Voglibose can be cited as an example of antihyperglycemic medicines used in the management of type 2 DM since they inhibit α-amylase/α-glucosidase, thereby leading to effective regulation. However, a number of oral hypoglycamic medications have a variety of adverse effects, including weight gain, gastrointestinal distress, hypoglycemia, cardiovascular problems, etc. [14,15]. Since the 1990s, acarbose has been used in clinical settings because of its ability to inhibit α-glucosidase in the lumen of the small intestine. Its drawbacks, however, include a high incidence of gastrointestinal adverse effects, such as diarrhea, bloating, and gas [16].

The class of thiazolidines is one of the most well known in the heterocyclic hybrid domain due to its broad range of biological actions, including glitazones. The structural foundation for the substituted analogs of its third and fifth, which show strong antidiabetic benefits, is the thiazolidinedione (TZD) ring system, which includes five members. At positions two and four, it contains carbonyl groups [17,18]. Various replacements on N-3 and C-5 positions are possible in the unique heterocyclic scaffold 2,4-TZD. Knoevenagel condensation with various arylidene moieties at the C-5 position yielded the most optimistic results. However, with the availability of moderate bases, the acidic nature of N-H facilitates the replacement of several alkyl/acetyls/benzyl halides at the N-3 position [19]. Thiazolidinediones are an attractive pharmacophore to the pharmaceutical and medicinal chemist. The compounds of thiazolidinedione are showing many different pharmacological actions, such as anti-Alzheimer, antibiotics, antimycotic, antiviral, anti-neoplastic, immunosuppressants, and hypoglycemic agents [20,21].

**Figure 1:**
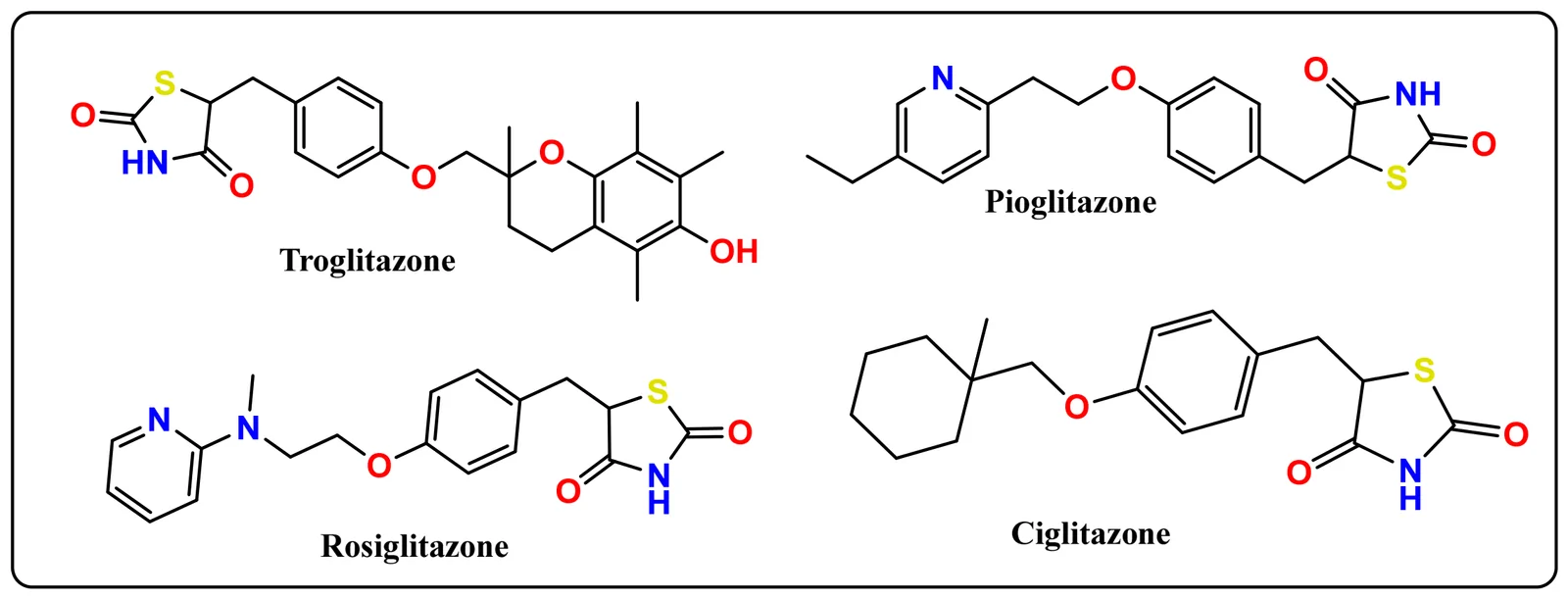
Chemical structures of the clinically approved thiazolidinedione antidiabetic drugs.

In treating type 2 diabetes, thiazolidinediones (TZDs), also called “glitazones”, can help with insulin resistance and blood sugar control [22]. Numerous advancements have occurred in the development of drugs that use glitazones, which started in the year 1982 when glitazone was first used to treat high blood sugar. Since then, other glitazone medications have been discovered, including Troglitazone, Pioglitazone, Rosiglitazone and Ciglitazone, which are shown in Figure 1 [23]. Recent studies suggest that rosiglitazone may harm the cardiovascular system and pioglitazone may cause bladder cancer. Even though there are some glitazone drugs like troglitazone and englitazone that have been discontinued for medical treatment due to their negative attributes, the shortcomings of a few of the currently available glitazones stress the importance of the development of novel drugs. As a result, there is an increasing demand for effective diabetes medicines for type 2 DM [24].

The current work aims at designing a set of thiazolidinedione derivatives (**D1**–**D30**) based on the previously provided information. To discover an effective antidiabetic compound, the designed compounds were subjected to screening, employing in silico techniques like docking, ADMET prediction, etc. The compounds were selected for screening among the top six compounds, which were chosen based on their pharmacokinetic parameters and binding affinities. MD was carried out on the top three compounds. The in vivo study was conducted using *Drosophila melanogaster*, and the recently created substances underwent in vitro inhibitory assays on the basis of their potential for antidiabetic drugs. The overall objectives of this research work include identifying effective TZD compounds with lesser side effects when compared to the standard drug acarbose.

## 2. Results and Discussion

### 2.1. In Silico Studies

Analysis of the lead compounds **D1**–**D5** via in silico Lipinski’s rule of five screening confirmed that all candidates align with established drug-likeness criteria. Each compound maintained a molecular weight under the 500 Da limit, a lipophilicity profile with a Log *p* value below 5, and an appropriate hydrogen-bonding capacity. These results suggest that the series possesses the necessary physicochemical foundation for favorable oral absorption and systemic permeability. This implies that the molecules exhibit favorable physiochemical and drug-likeness properties. These lead compounds possessed optimal Log *p* values (2.09–3.16) indicating good hydrophobicity and lipophilicity, a factor necessary for proper movement through membranes. In addition, the number of hydrogen bond donors (2–3) and acceptors (7–8) were optimal, thus implying that the drug molecules have favorable properties necessary to be effective when ingested orally. It is worthy of note that none of the compounds violated Lipinski’s rule, thus exhibiting good properties required for effective oral drugs. The standard drug molecule Acarbose, on the other hand, had three violations of Lipinski’s rule as shown in Table 1. These violations include high molecular weight and too many hydrogen bond formations, thus decreasing permeability and oral absorption.

### 2.2. Predicted ADME Parameters

The predicted ADME (absorption, distribution, metabolism, excretion) parameters from QikProp give clues about how the lead compounds (**D1**–**D5**) behave pharmacokinetically. The lead compounds demonstrated overall good pharmacokinetic (ADME) profiles compared to the stand-alone drug acarbose. The %HOA (percent human oral absorption) for **D1**–**D5** ranged from a low of 67.55% (**D3**) to a high of 88.95% (**D1**) suggesting moderate-to-high levels of oral absorption, while acarbose had a measured value of 0%. Therefore, acarbose has poor bioavailability. The water solubilities of the lead compounds (QPlogS) as expressed in the range (−4.2 to −5.4) have values within an acceptable range, indicating the lead compounds will have sufficient solubility to act as drugs. The permeability of the lead compounds (**D1**–**D4** and **D5**) as shown using the Caco-2 cell and MDCK model exhibited moderate levels of permeability (**D1**–**D3** and **D5**), with **D4** exhibiting a significantly lower level of permeability than the other compounds studied. Acarbose exhibited an extremely low level of permeability in both permeability models when measured, thus limiting the absorption of acarbose in the intestines. In general, the lead compounds all fall into an acceptable range (−1.4 to −2.4), as measured by their ability to cross the blood–brain barrier (QPlogBB), which suggests that the lead compounds will diffuse into the central nervous system (CNS) at a very low level, thereby decreasing the potential for CNS side effects. Protein-binding (QplogKHSA) values were also within the normal range, indicating balanced distribution in systemic circulation. The number of predicted metabolic reactions (#metab) for **D1**–**D5** ranged from three to five, suggesting moderate metabolic stability, whereas Acarbose showed a higher value (14), showing extensive metabolism. Permeability through the skin (QPlogKp) values of the lead compounds were within acceptable limits, further supporting their favorable pharmacokinetic properties shown in Table 2. The ADME analysis suggests that the lead compounds possess significantly better absorption, permeability, and overall pharmacokinetic profiles compared to Acarbose, highlighting their potential as promising drug candidates for further studies.

### 2.3. Drug-Likeness and Toxicity Studies

Compounds **D1** to **D6** were assessed with the Osiris Property Explorer software-V2 for their drug likeness and toxicity to help in evaluating their potential to be developed as drugs. Most of the compounds demonstrated favorable properties when compared with the reference drug Acarbose. The Clog *p* values for the compounds (1.2 to 2.84) indicate balanced lipophilicity, an important property in allowing for adequate permeability across membranes, which would be necessary for absorption. The solubility values for the compounds (−3.49 to −4.62) suggest that they have moderate aqueous solubility and are likely to be taken up by the body at an adequate level. By contrast, the reference drug has a very high negative value for Clog *p* (−7.91) and has higher solubility, suggesting an excessive amount of hydrophilicity which could limit its ability to move across membranes shows in Table 3.

Generally speaking, all four compounds **D1**–**D3** and **D5** had positive drug-likeness scores indicating they all resemble drugs according to their structural features. **D1** had the highest score of 6.25. This score is indicative of having drug-like characteristics. However, compound **D4** has a negative score (−9.26) indicating its structure is not conducive to being classified as a drug. The drug scoring of **D1**–**D3** all had high scores (0.79), which suggests they are the best candidates when compared with all of the other compounds on the basis of inherent characteristics of drugs (i.e., physical chemical properties, solubility, etc.). Since each compound has some level of toxicity, there were low or no toxicity levels for compounds falling within the green range for tumorigenic, mutagenic, irritant or reproductive. In contrast, compounds **D4** and **D6** demonstrated comparatively higher toxicity results than compounds **D1**–**D3** as indicated by red coloration, which may indicate adverse effects. The results demonstrate that compounds **D1**–**D3** and **D5** have potential to be classified as drug likeness and have superior physicochemical characteristics, and therefore will be considered for further experimental development and validation.

### 2.4. Molecular Docking Studies

**Figure 2:**
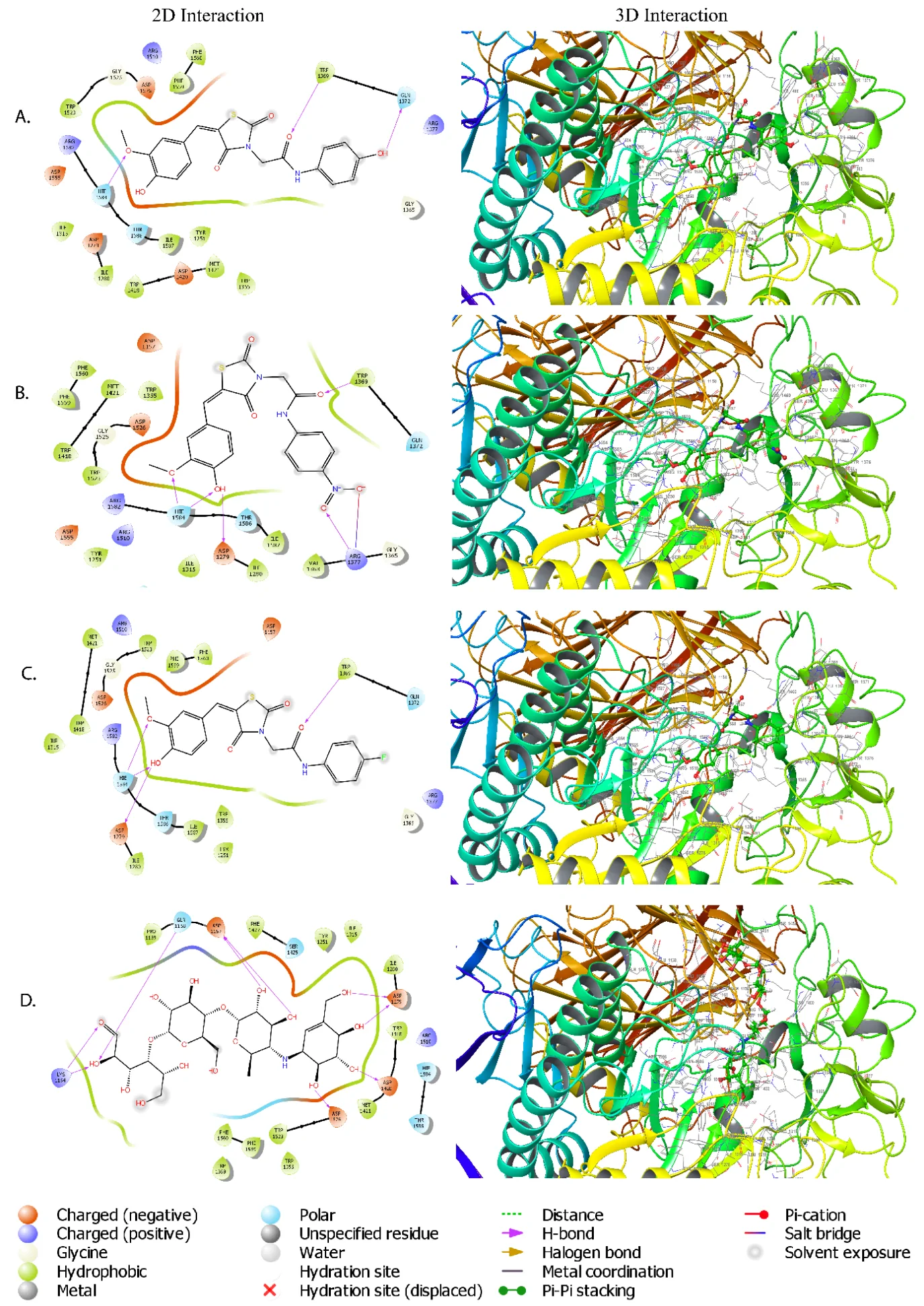
(**A**) 2D and 3D interaction of compound **D1** with 3TOP; (**B**) 2D and 3D interaction of compound **D5** with 3TOP; (**C**) 2D and 3D interaction of compound **D4** with 3TOP; (**D**) 2D and 3D interaction of acarbose with 3TOP.

The binding interaction of selected 2,4-thiazolidinedione derivatives (**D1**, **D4**, and **D5**) with the target protein 3TOP was evaluated using molecular docking analysis and compared against Acarbose which is the standard. The binding energies for all lead compounds indicated that they all showed excellent binding affinity for the binding site of the target protein. The compound with the lowest binding energy was **D1** (−7.74 kCal/mol), followed by **D5** (−7.39 kCal/mol) and **D4** (−7.34 kCal/mol). In contrast, Acarbose had a significantly lower binding energy (−10.73 kCal/mol), showing a very promising interaction with the target enzyme (Table 4). Hydrogen bonding plays a crucial role in ligand–protein stability. **D4** formed the highest number of hydrogen bonds (five), followed by **D1** and **D5** (three each), interacting with key amino acid residues such as Trp1369, Asp1279, Gln1372, Arg1377, and Hie1584. Acarbose, in comparison, formed nine hydrogen bonds with multiple active site residues, contributing to its stronger binding affinity shown in Figure 2. The interacting residues observed for the lead compounds, including Trp1369, Asp1279, Asp1526, and Arg1582, are important for stabilizing ligand binding within the active pocket. Although no π–π stacking interactions were observed, several hydrophobic and polar interactions contributed to the overall stability of the ligand–protein complex.

**Figure 3:**
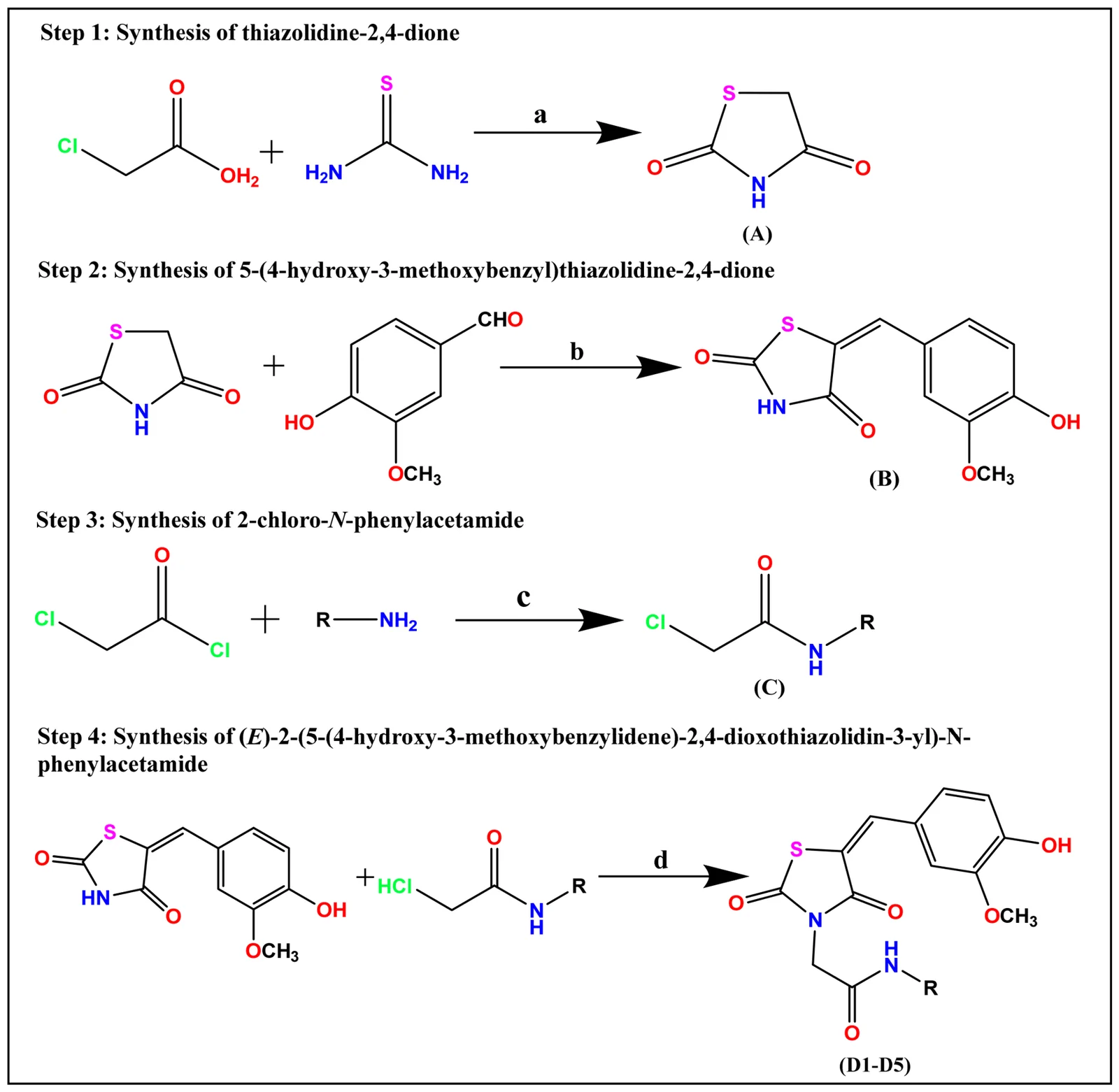
Synthetic scheme reagents and conditions: (**A**) H_2_O, stir for 1 h, HCl; (**B**) ACN, glacial acetic acid, piperidine, reflux at 60–70 °C, 3 h, cooled to RT, conc. HCl; (**C**) DCM, TEA, stir overnight, 1000 rpm, H_2_O, anhydrous Na_2_SO_4_; (**D1**–**D5**) ethanol, K_2_CO_3_, reflux, 6 h, ice-cold water. **D1**: 4-OH, **D2**: 3-OH, **D3**: 2-OH, **D4**: 4-NO_2_, **D5**: 4-F.

The 2D and 3D interaction analyses further confirmed that all compounds fit well within the active site of the target protein and formed interactions that were stable with key residues, similar to the standard drug shown in Figure 3. The binding affinity of the lead compounds is slightly lower than Acarbose; their stable interactions and favorable binding patterns suggest that **D1** and **D5**, in particular, may serve as promising inhibitors and potential candidates for further optimization and experimental validation.

### 2.5. MM-GBSA Binding Free Energy Studies

According to the results of the energy calculation study (by use of the MM-GBSA method), the binding free energies of N-[4-(benzyloxy)-3-methoxyphenyl]succinimide, H2N-1-(benzylcarbamoyl)-3-bromo-2-methyl-5-substituted-3H-pyrrolizine, N-[4-(benzyloxy)-3-methoxyphenyl]succinimide and N-[4-(benzyloxy)-3-methoxyphenyl]succinimide measured at a constant temperature are within the range of order 4 kcal.mol1. The comparison of lead compounds against Acarbose demonstrated that lead compounds displayed a more positive energy result of ΔGbind (+1.0 kcal/mol) than Acarbose for lead compounds and suggested Acarbose has significantly inferior electrostatic interaction compared to the lead compounds. This is in line with previous studies that suggested the interaction was highly reliant on lipophilic van der Waals sites. The energy decomposition results suggest that the most important contributors to the binding stabilization of the three lead compounds were hydrophobic (lipophilic) and van der Waals energy contributions (ΔGbind Vander). This demonstrates an abundance of hydrophobic interactions between the lead molecules and the target protein 3TOP. The electrostatic contributions for lead compounds **D1** and **D2** increased binding stabilization due to their higher electrostatic interaction with the target protein than other lead compounds (**D3**, **D4** and **D5**). Therefore, binding stability for lead compounds will be created through a combination of van der Waals and hydrophobic energies as shown in Table 5.

The contributions of hydrogen-bonding energies were moderate, contributing to the overall stability of the complexes through their supportive nature. Covalent (internal) energy values were positive for all compounds, indicating that, upon binding, the ligands experienced internal strain (due to the energetic cost of ligand–protein binding) so that the internal energy of the ligands was “stressed” (energy) by binding to the target. MM-GBSA calculations point to **D1** in particular having a more stable and energetically favorable interaction with the target compared to other lead compounds. The results support the molecular docking analyses and provide further evidence that the compounds show promise as candidates for further drug development, particularly **D1**.

### 2.6. Molecular Dynamics Simulations

**Figure 4:**
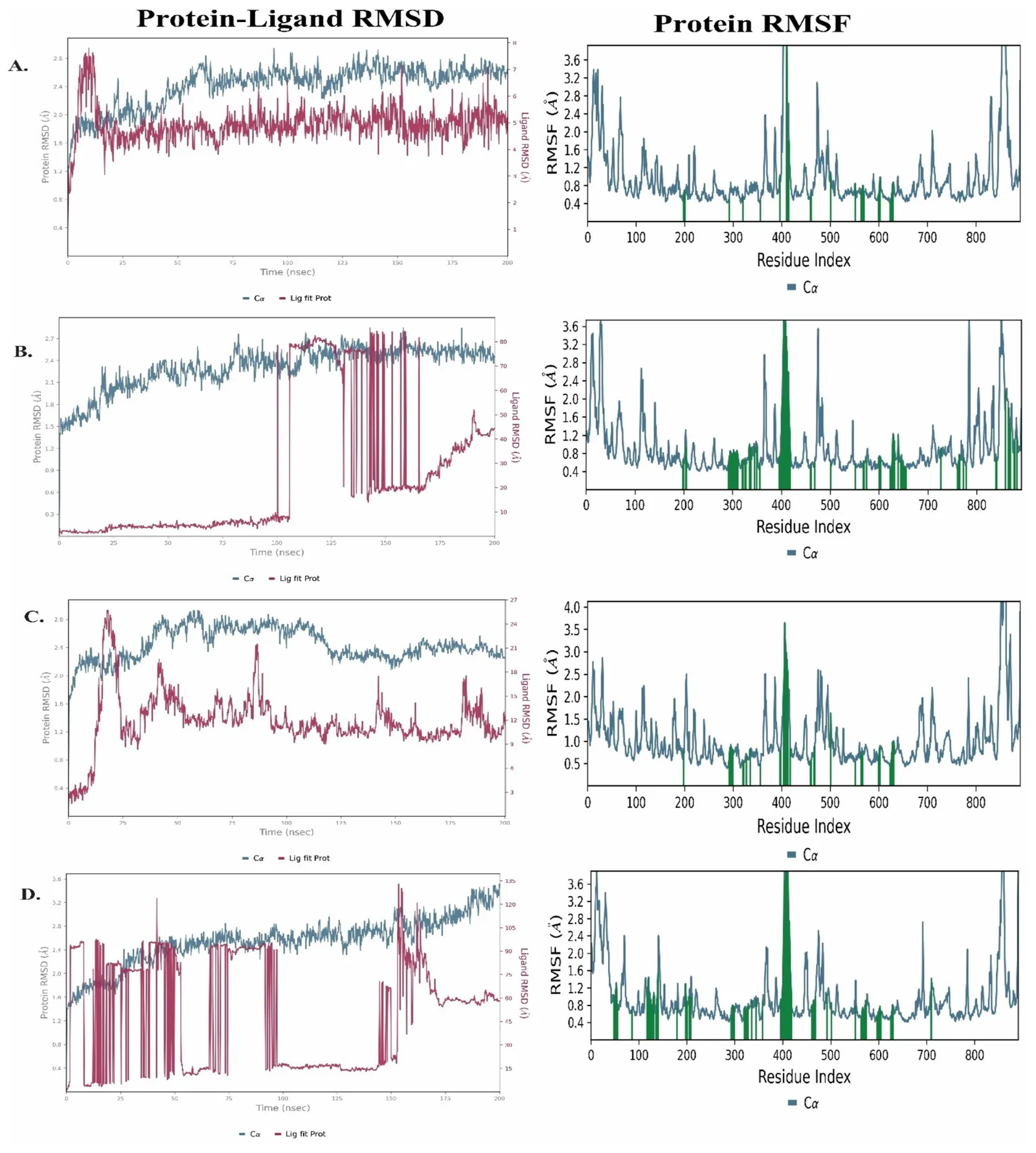
Analysis of **RMSD** and **RMSF** of 3TOP protein with ligand at 200 ns. (**A**) RMSD (protein RMSD is shown in gray while RMSD of compound **D1** is shown in red), protein RMSF, (**B**) RMSD (protein RMSD is shown in gray while RMSD of compound **D5** is shown in red), protein RMSF, (**C**) RMSD (protein RMSD is shown in gray while RMSD of compound **D4** is shown in red), protein RMSF, (**D**) RMSD (protein RMSD is shown in gray while RMSD of standard acarbose is shown in red), protein RMSF.

Simulations of the 3TOP–ligand complexes over 200 ns showed that in all cases, the ligands bound stably and displayed considerable structural stability while binding to the protein. The protein RMSD profiles revealed an initial equilibration period with stable trajectories (~2.0–3.0 Å), which shows that the binding of ligands to the target did not cause gross changes in the conformation of the protein, even during the extended duration of the simulation shown in Figure 4.

The **D1** complex was ascertained to have highly stable behavior over the 200 ns simulation period and only had fewer fluctuations in the RMSD values of the ligand during the simulation period (indicating a strong interaction at the active site). The **D5** complex was also found to exhibit stable protein behavior with a moderate degree of flexibility of the ligand that likely allows for adaptive binding, but does not adversely affect the integrity of the complex. Additionally, the **D4** complex was discovered to be stable over the entire simulation time, indicating that it has strong potential for appropriate accommodation within the binding pocket throughout the simulation timeline. The standard acarbose complex was found to behave as expected in terms of dynamic flexibility due to its larger molecular structure, but was found to remain stably bound to the active site. This gives additional confidence in the simulation system as being a valid representation of the actual protein/ligand complex. The RMSF analysis indicated that the majority of the residues had low fluctuations, suggesting that the protein backbone was stable during the simulation time period. The loop regions exhibited slightly higher flexibility, which is customary for loop regions; this greater flexibility can be advantageous with respect to facilitating the dynamics associated with ligand–protein interactions. Also, the key residues that comprise the active site were found to have not experienced any significant fluctuations during the course of the simulation, indicating that the ligand was stably engaged in the active site throughout the entire simulation. The extended 200 ns simulation indicated that all compounds were found to form stable complexes with 3TOP and are thermodynamically favorable for complex formation with 3TOP, with **D1** and **D5** demonstrating superior dynamic stability to the standard complex. Therefore, these compounds are likely to be the most effective at being α-amylase inhibitors and would support their use in the continued development of new drugs.

### 2.7. Protein–Ligand Contact Analysis

**Figure 5:**
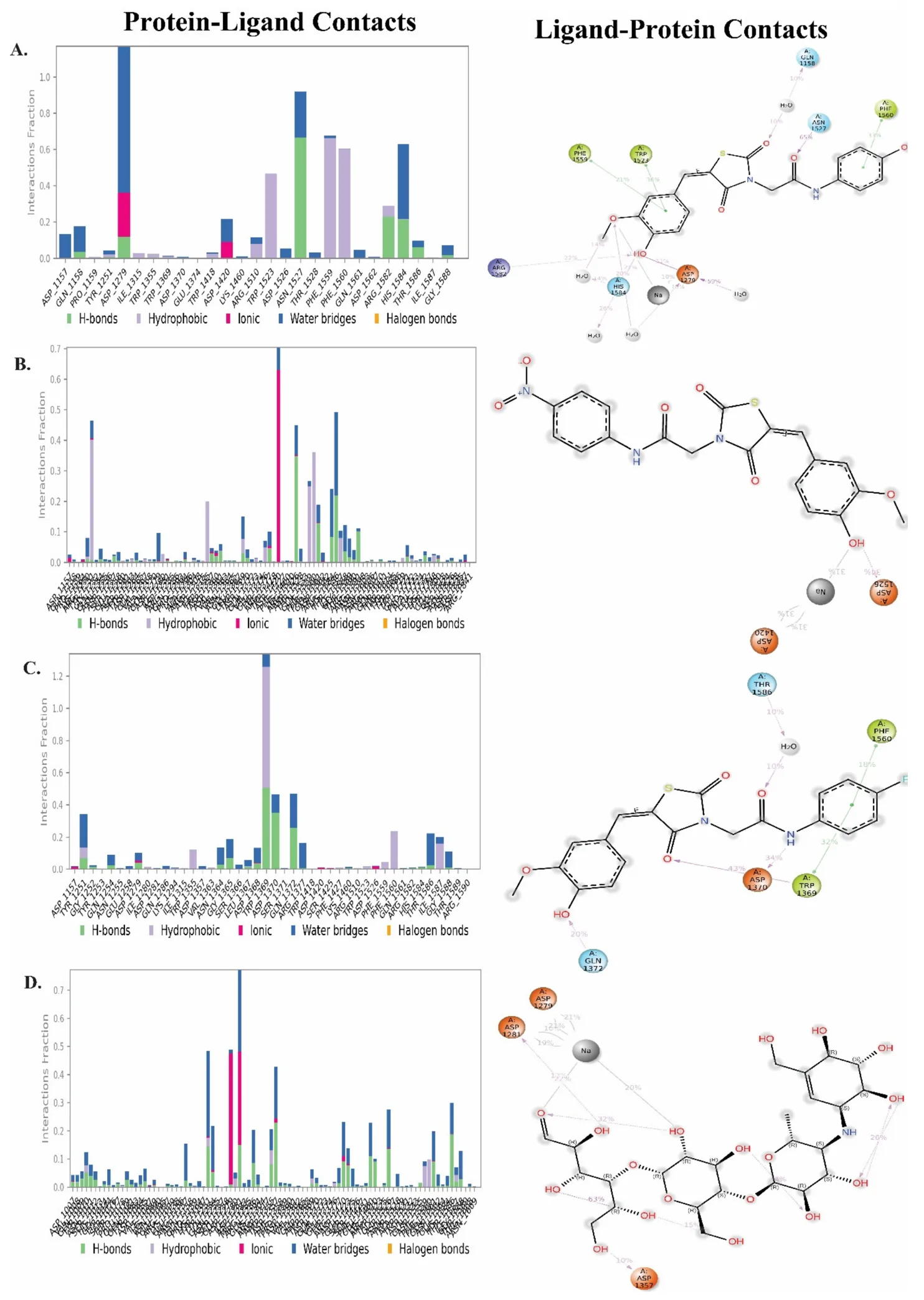
(**A**) Molecular dynamics (MD) of trajectory-based protein–ligand contact analysis and 2D interaction profile for compound **D1**; (**B**) MD-derived contact analysis and 2D interaction mapping for compound **D5**; (**C**) MD trajectory contact evaluation and 2D binding site analysis for compound **D4**; (**D**) MD-based contact analysis and 2D interaction diagram for the reference drug, acarbose.

Analysis of the MD trajectories shows that there is a stable and consistent interaction between 3TOP and each of the ligands that were examined. The interactions were hydrogen bonds, hydrophobic contacts, and water bridges which were preserved throughout the simulation period, indicating that the ligands fit well into the active site of the target. In the case of the **D1** complex (Figure 5A), it showed very strong and persistent interactions with the binding site of the protein, especially the H-bonding to the key residues at the active site, which was stable for a long portion of the trajectory. This indicates that there is a high amount of binding stability. For the **D5** complex (Figure 5B), it has also shown stable interaction patterns and evidence of both directly hydrogen-bonding interactions as well as water-mediated interactions, which indicates that there is a high degree of binding adaptability and retention in the pocket. The **D4** complex (Figure 5C) had moderate interaction frequencies, fewer persistent hydrogen bonds, and greater reliance on hydrophobic interactions. Even though the ligand was still bound, the moderate interaction stability suggests that there is somewhat of a decrease in the binding strength. The standard acarbose molecule illustrated by Figure 5D forms a large network of hydrogen bonds and water bridges, indicating its strong/specific interaction to the site, and therefore serves as a promising reference point for further comparison. **D1** and **D5** both show binding interactions to 3TOP that are equivalent to acarbose, while **D4**’s binding stability is moderate. The results of these studies further confirm that **D1** and **D5** are potential inhibitors of 3TOP and are consistent with the stability observed during MD and biological activity.

### 2.8. In Vitro Studies

**MTT Assay**

**Figure 6:**
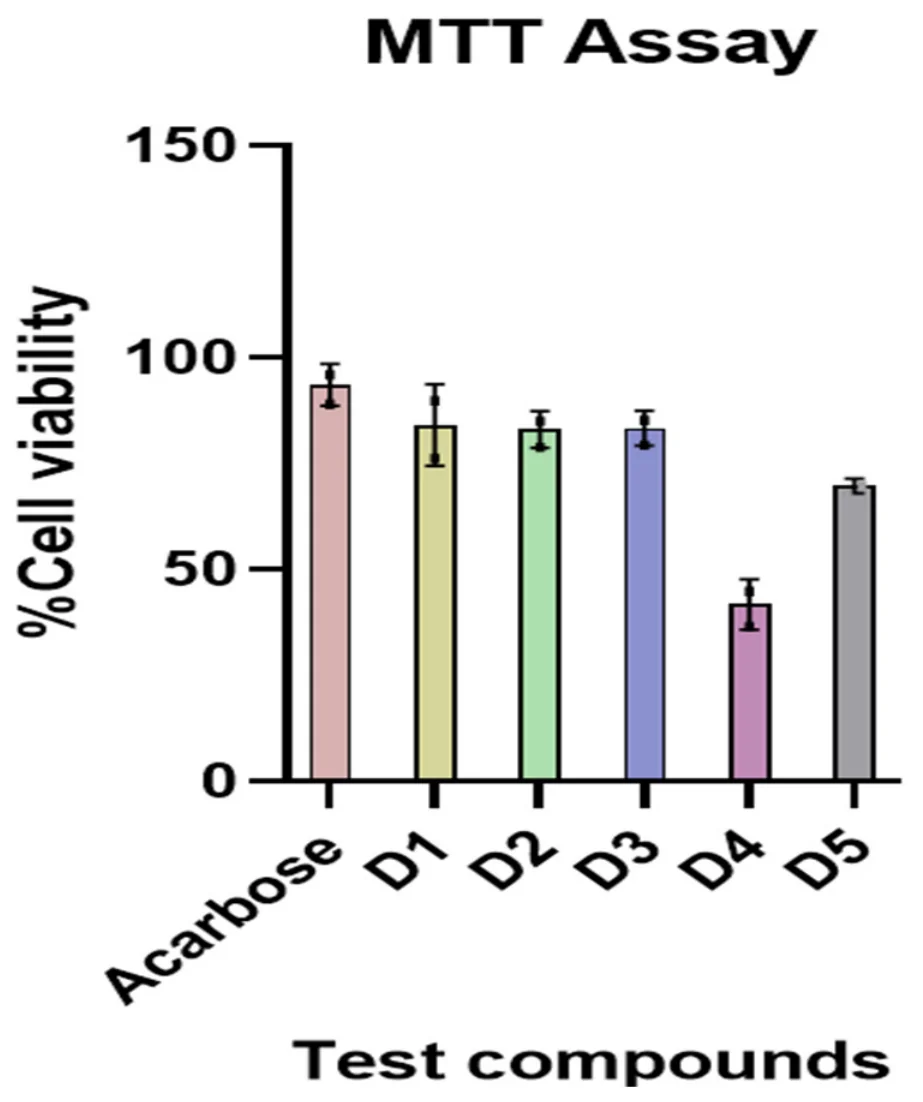
Values are mean ± SD from triplicate determined (n = 3). The percentage of cellular toxicity of different compounds (**D1**–**D5**) at a conc of 200 μg/mL.

Cytotoxicity of the 2,4-thiazolidinedione derivatives (**D1**–**D5**) was measured through a mitochondrial metabolism (MTT) assay using 200 µg/mL of each compound. Most of the derivatives demonstrably had excellent cell viability (>70%), suggesting that most of the compounds had low-to-moderate cytotoxicity at this concentration. **D2** and **D3** showed similar cell viabilities as acarbose, suggesting that both compounds have an acceptable safety profile and do not significantly inhibit cellular metabolic activity. **D1** had a good degree of cytocompatibility, but slightly lower than either **D2** or **D3**. The **D4** derivative had a markedly lower cell viability, suggesting that **D4** has a greater cytotoxicity than the other compounds; therefore, it is possible that structural aspects of this compound are affecting mitochondrial function and/or creating a stressful environment for the cells. **D5** exhibited moderate cytotoxicity, suggesting a dose- or structure-dependent effect. The results highlight that **D2** and **D3** are the safest candidates, while **D1** and **D5** present a balance between cytocompatibility and previously observed biological activity as shown in Figure 6. These findings, when correlated with in vivo efficacy in *Drosophila melanogaster*, support the selection of promising lead compounds for further optimization and detailed toxicity evaluation.

### 2.9. In Vitro Antidiabetic Activity

In vitro tests that looked at how well the compounds (**D1**–**D5**) could stop α-amylase and α-glucosidase were used to see how well they could work as antidiabetic drugs. Acarbose was used as the reference standard, and each compound was tested in a range of 10–50 μg/mL. We looked at how well each compound worked to stop something, and the IC_50_ values are shown in Table 6. Compounds **D1** (33.86 ± 2.1 μM) and **D5** (37.55 ± 1.7 μM) were good at stopping α-amylase in contrast to the reference compound. The results match the docking score that was given. The reported antidiabetic action is due to the availability of substituted groups at the fourth position of the phenyl ring. When compared to the standard, compounds **D1** (29.81 ± 3.2 M) and **D5** (32.43 ± 1.2 M) had strong activity in the α-glucosidase assay (Table 6). The more active compounds from in silico experiments were made by designing and testing a new group of thiazolidinedione derivatives. It was made and studied using the Knoevenagel condensation method. Newly synthesized molecules were tested for both in vitro and in vivo antidiabetic activity by targeting enzymes like α-amylase and α-glucosidase that are involved in breaking down starch, absorbing glucose, and controlling blood sugar levels. **D1** and **D5** exhibited promising inhibitory efficacy among the evaluated pharmaceuticals. These results suggest that these substances may be effective for diabetes treatment. Docking and simulation analysis further corroborated the rational design of antidiabetic medications.

### 2.10. In Vivo Antihyperglycemic Activity in Drosophila melanogaster Model

This study evaluated the antihyperglycemic potential of synthesized 2,4-thiazolidinedione derivatives using an in vivo *Drosophila melanogaster* model under high-sugar diet (HSD)-induced metabolic stress. The estimation of glucose levels in both female and male flies at two concentrations (300 ppm and 600 ppm) provides important insights into the dose-dependent efficacy and sex-specific responses of the tested compounds.

**Figure 7:**
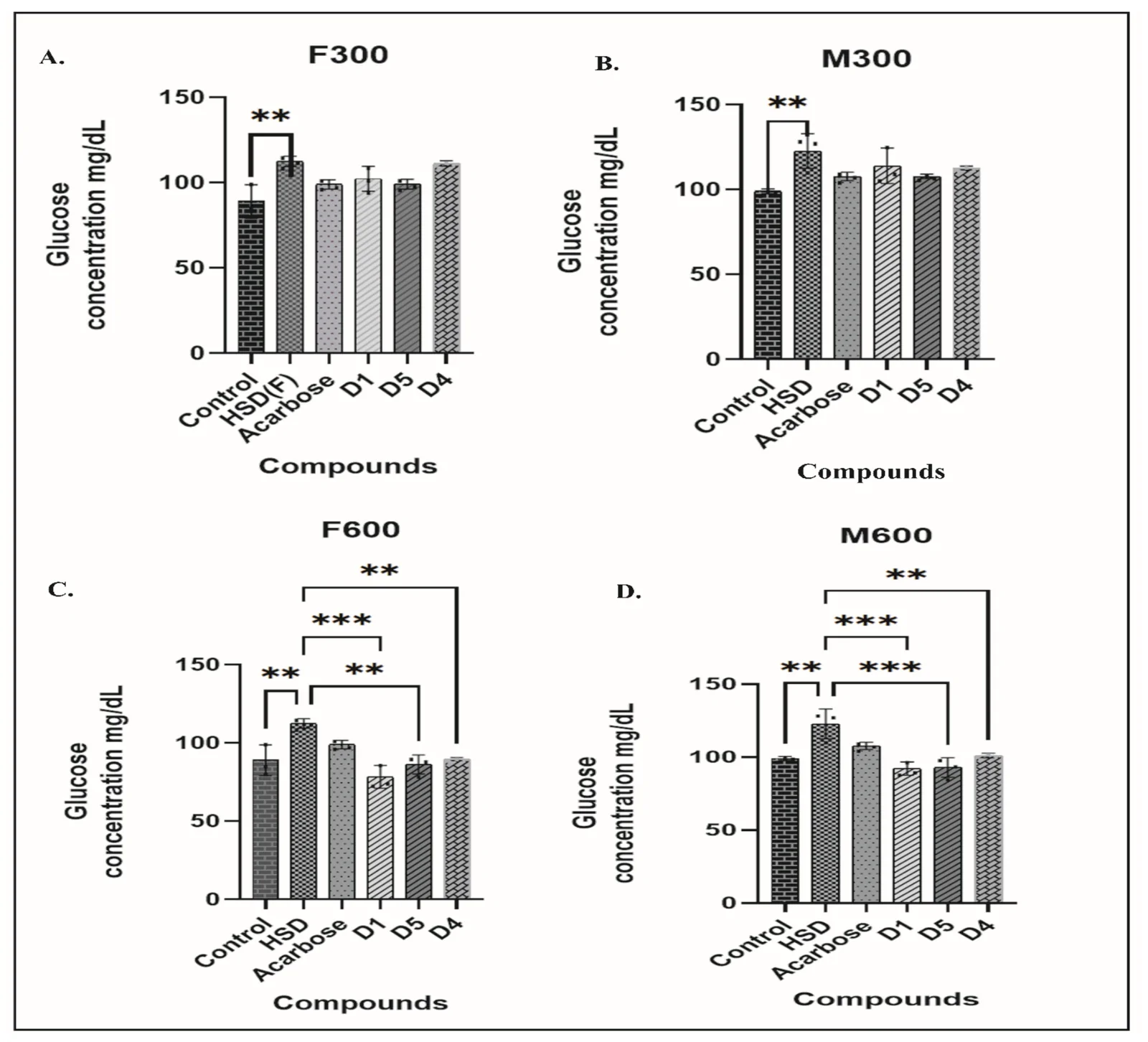
Estimation of glucose concentration in Drosophila model. (**A**) Glucose concentration of female flies at 300 ppm concentration, (**B**) glucose concentration of male flies at 300 ppm concentration, (**C**) glucose concentration of female flies at 600 ppm concentration, (**D**) glucose concentration of male flies at 600 ppm concentration. All the results expressed are the mean of three biological replicates (n = 3). ** *p* < 0.01 and *** *p* < 0.001.

The flies fed the high-sugar diet and treated with 300 parts per million (F300 and M300) had higher glucose concentrations than the control, thus supporting that the treatment group had developed hyperglycemia. Treatments with the standard hypoglycemic drug, acarbose, produces statistically significant reduction in glucose concentrations compared to the high-sugar diet, confirming its usefulness as a model. The rest of the test compounds (**D1**, **D5**, and **D4**) also produced moderate reductions in glucose, with values approaching those of the standard group, although not quite as dramatic as compared to the higher-dosage treatments. These results indicate that despite the intrinsic antihyperglycemic properties of the compounds, their efficacy at lower doses may be decreased. When examined at the higher concentration of 600 ppm (F600 and M600), the reductions in glucose concentrations for both males and females were more pronounced, as shown in Figure 7. Compounds **D1** and **D5** produced significant reductions in glucose concentrations that were equivalent to, or greater than, acarbose in females. The statistically significant findings (*p* < 0.01 and *p* < 0.001) between the treated and high-sugar diet groups show that the potency of the compounds is greatly increased at the higher dose.

This dose-dependent improvement suggests stronger inhibition of enzymes such as α-amylase and α-glucosidase that metabolize carbohydrates, resulting in reduced glucose availability. A comparison between sexes revealed slight variations in response, with female flies generally showing a marginally better response to treatment than males. This may be attributed to differences in metabolic regulation, hormonal influence, or energy storage mechanisms in *Drosophila melanogaster*. However, the overall trend of glucose reduction remained consistent across both sexes, reinforcing the reproducibility of the findings. The current data indicate that the thiazolidinedione derivatives that were evaluated showed the capacity of these compounds to reduce hyperglycemia in vivo from HSD, supporting the assumption that they work as dual inhibitors for each enzyme. Also, the improved efficacy at 600 ppm supports the theory that there is likely a “maximum” therapeutic level for the thiazolidinedione derivatives to provide maximum biological response. These results provide convincing evidence that both of the compounds that were synthesized (**D1** and **D5**) have demonstrated considerable antihyperglycemic capability in the in vivo system. In addition, when these results are considered together with the previous in silico and in vitro studies done, the compounds have been demonstrated to have considerable potential to be lead candidates for further development in the area of antidiabetic therapy. Continuing to develop our understanding of the detailed mechanism by which the compounds exhibit their effects, toxicity profiling and validating their clinical application using higher-order animals would be appropriate next steps in the development of these compounds.

**Structure–Activity Relationship (SAR)**

The biological activity of the designed 2,4-thiazolidinedione derivatives depended on the type and position of substituents on the N-phenyl ring. Compound **D1** (para-OH) had the activity and binding affinity due to the hydrogen-bonding interaction with the amino acid residues in the active site. Compound **D5** (para-F) had activity due to the improvement in hydrophobic interactions and lipophilicity. Compounds **D2** (o-OH) and **D3** (m-OH), however, were less potent, indicating the unfavorable binding pose when moving the hydroxyl substituent away from the para position. Compound **D4** (p-NO_2_) had the least activity due to the destabilizing effect of the highly electron-withdrawing nitro group on binding. The para-substituted and electron-donating or slightly lipophilic groups were more preferred for α-amylase/α-glucosidase inhibition in the following order: **D1** > **D5** > **D2** = **D3** > **D4**.

## 3. Materials and Methods

Sigma Aldrich and Loba Chemie in Mumbai, India, supplied the chemicals and reagents. We used n-hexane and ethyl acetate as a solvent system with a ratio of 2:3. Thin-layer chromatography (TLC) investigated the homogeneity of the substances. We employed silica GF254 plates that had been coated, making the dots visible in the UV chamber. We determined the melting points with a digital melting point instrument from Equiptronics (Mumbai, India), and the results remain unadjusted. To record the FT-IR spectra in cm^−1^, an Alpha Bruker IR spectrometer (Billerica, MA, USA) was used. We used the Jeol ECZ Four hundred FT-Nuclear Magnetic Resonance spectrometer (Tokyo, Japan) operating at 400 MHz to record ^1^H-NMR spectra, and the same spectrometer running at 100 MHz for ^13^C-NMR spectra. For the internal standard, we used trimethylsilyl (TMS) in δ ppm. We recorded mass spectra using the Shimadzu LC/MS-8030 series (Kyoto, Japan).

### 3.1. In Silico Analysis

**Docking of Molecular Structures**

For assessing the interactions of the synthesized molecules with their target receptors in the biological system, in silico molecular docking simulations were carried out. These tests were performed to get insights into the mechanism of action, that is, the mode of binding and molecular orientation in the active site. The docking study was done by employing Schrödinger Suite (2020-4) [25].

**The Preparation of Ligands**

The novel series of thiazolidinedione derivatives (**D1**–**D50**) were designed using Chemdraw 22.0.0 software. Following the conversion of the chemical library from 2Dimension to 3Dimension formats, the module for Ligprep of the Schrodinger suite-2020-4 was utilized to optimize geometry, select the bond order, and generate the ligand ionization state. The force field OPLS-3 was used to reduce the ligands’ energy. For docking investigations, we used these optimized ligands. To achieve higher docking scores, the docking structures need to accurately represent the ligand structures as they form during a protein–ligand interaction. Optimizing the reset of the geometric patterns should come first. The docking tools change the ligand’s torsional coordinates. This implies that all required standard conditions must be met by the structures that are inserted into the docking system. Docking studies were conducted with the ligands that were modified to meet all the requirements [26].

**Preparing Protein**

The RCSB PDB (available online: https://www.rcsb.org/) provided the Human Maltase-Glucoamylase with acarbose in complex (PDB ID:3TOP, 2.88 Å). The protein was prepared using the Schrodinger suite 2020-4 [27]. Missing hydrogen atoms were added, suitable bonding configurations were provided, potential metal contacts were addressed, and water molecules that were within 5 Å of heterogeneous groups were eliminated in order to prepare the protein. Through sample orientation, hydrogen bonds were optimized and all of the hydrogen atoms in the polar groups were visible.

**Protein Docking with Ligands**

In the molecular docking technique, the grid that was formed would specify the exact area in the targeted protein where the ligand would bind to the protein. The interface of the Maestro Glide Tool was used to generate the grid. OPLS3e was applied in the grid settings. After the specification of the receptors’ folders through LigPrep, the receptors were chosen in the Glide method with the flexibility in the XP mode applied. The resulting binding interactions were ordered based on calculated scores inclusive of grid score, proprietary Glide score and internal energy strain. A pose-viewer was also used to visualize these results in a variety of structural output formats. The Glide score was used in prioritizing the ligands as well as in making a prediction of binding affinity. The molecular docking study of α-amylase and α-glucosidase inhibitors is carried out using the extra precision (XP) mode of the current docking tool [28].

### 3.2. Calculating of Prime/MMGBSA

For the thermodynamic stability of the protein and ligand complexes, the free energy of binding was determined using the Prime MM-GBSA package in the Schrodinger software suite version 2020-4. It offered the possibility of the quantitative evaluation of the affinity of the newly designed compounds towards the target protein. After the first stage of docking, when the complexes were obtained via the docking procedure in the extra precision (XP) mode, the resulting structures were subjected to energy minimization to further improve the accuracy of the binding study. Energy minimizations were performed for the complexes using the OPLS3e force field and the VSGB 2.0 continuum solvent model [29].

### 3.3. Molecular Dynamics (MD) Simulations

MD simulation studies were employed to ascertain the interactions between the common acarbose and the synthetic ligands (**D1**, **D4**, **D5**) and the targets α-amylase and α-glucosidase. The computational simulations utilized the OPLS-2005 force field (Optimal Potentials for Liquid Simulations) and the TIP4P water model for system solvation. To maintain the necessary separation between the protein and the buffer, an orthorhombic water box was established, ensuring a 10A^0^ buffer zone between the protein’s atoms and the edges of the simulation box. The systems were neutralized and any overlapping water molecules were eliminated using Na+ ions. Energy estimates were made using the force field from OPLS-2005. The temperature was maintained at 300K with an incorporation step of 2.0 fs. To monitor the stability of proteins while they move naturally, the root mean square deviation (RMSD) was calculated. Compounds **D1**, **D5**, **D4** and acarbose were selected based on Glide dock XP values, and the simulations were performed for 200 ns using the Maestro 12.6 Desmond panel. Throughout the simulation, 1000 frames were produced and the simulation was recorded at 20 ps intervals [30].

### 3.4. Assessment of the ADME Toxicity of Compounds (***D1***–***D5***)

The Qikprop module was employed to predict the ADME features of each new molecule in order to find out the drug-like qualities. The molecular descriptor and ADME profile were predicted by Maestro Schrödinger’s QikProp module, version 5.4 [31].

**Predicting Drug Likeness**

When using chemical structures, the OSIRIS Property Explorer frequently calculates a variety of important parameters concerning drugs for the search structure. Values and color codes have been provided for the predicted results. Some of the parameters for which the computations were carried out include TPSA, drug score, molecular weight fragment-based drug likeliness, toxicity, log S calculation, clog *p* calculation, etc. [32].

### 3.5. Thiazolidine-2,4-dione Synthesis (A) 

A total of 5 mL of water was used to separately dilute thiourea (0.02 mol) and chloroacetic acid (0.02 mol). The vessel’s contents were transferred into a conical flask and then agitated for about an hour at 400 rpm. The 6 mL of concentrated HCl is then added, resulting in the formation of a precipitate. Alcohol was used to filter, purify, and recrystallize the white substance that had settled out (Figure 2) [33].

**Synthesis of 5-[4-hydroxy-3-methoxybenzyl]-thiazolidine-2,4-dione (B):**

A mixture of thiazolidine-2,4-dione (0.01 mol) and 4hydroxy-3-methoxybenzaldehyde (0.01 mol) in 8 milliliters of aceto-nitrile was prepared. This was followed by refluxing at 60–70 °C for three to four hours with glacial acetic acid, and piperidine in small quantities for catalysis. The reaction was continuously monitored using thin-layer chromatography. When it was done, the reaction mixture was left to cool to room temperature and 1M HCl was added until precipitation was formed. Then, the cold water was added to the mix and filtered and dried. The final step was purifying the crude product by recrystallizing it in ethanol [34]. 

**Synthesis of Substituted 2-chloro-N-aryl acetamide Derivatives (C):**

In a cold environment, a base (triethylamine) and a substituted aryl amine (0.01 mol) in dichloromethane interacted with chloroacetyl chloride (0.01 mol). The reaction mixture was agitated all night at room-temperature conditions. Water was added after it was finished to help the organic layer separate. To the separated organic layer, anhydrous Na_2_SO_4_ was added to form the crude product, and filtered to remove the organic solvent. Ethanol was used to carry out further purification [35].

**Synthesis of 2,4-thiazolidinedione Derivatives (**D1**–**D5**):**

When 5-[4hydroxy-3-methoxybenzyl]-thiazolidine-2,4-dione (0.01 mol) and K_2_CO_3_ were combined, 2-chloro-N-aryl acetamide (0.01 mol) was substituted in DMF inside a round-bottomed flask (RBF). The mixture was refluxed for four to six hours, roughly. TLC, employing n-hexane and the mobile phase, ethyl acetate (9:1 *v*/*v*), was used to monitor the reaction’s progression. The reaction mixture was finished, left to cool, and then added to cold water. Absolute ethanol was utilized to filter, dry, and recrystallize the solid product that resulted [36].

**(*E*)-2-(5-(4hydroxy-3-methoxybenzylidene)-2,4-dioxothiazolidin-3-yl)-N-(4-hydroxyphenyl)acetamide (**D1**):**

Molecular formula: C_19_H_16_N_2_O_6_S, yellow solid, yield: 48.5%, FT-IR (cm^−1^): 3433 (N-H), 2762 (C-H), 1659 (C=C), 1592 (C=O); ^1^H-NMR (400 MHz, DMSO-d_6_, δ, ppm): 12.34 (O=C-NH-), 10.01 (-OH), 9.45 (-OH), 7.43 (ar-H), 7.14–7.13 (d, ar-H, 2H), 7.02–7.00 (m), 6.89–6.87 (d, ar-H), 4.83 (s, CH_2_), 3.83 (s, CH_3_); ^13^C-NMR (100 MHz, DMSO-d_6_, δ, ppm): 176.8, 172.8, 158.8, 156.3, 149.8, 148.2, 137.1, 135.0, 134.8, 130.9, 129.2, 128.5, 128.5, 124.0, 123.3, 119.7, 118.6, 63.4 and 55.5; the LC-MS (*m*/*z*) value for C_19_H_16_N_2_O_6_S is 400.41, found to be 400.50 (M^+^) (Figures S1–S4).

**(*E*)-2-(5-(4hydroxy-3-methoxybenzylidene)-2,4-dioxothiazolidin-3-yl)-N-(3-hydroxyphenyl)acetamide (**D2**):**

Molecular formula: C_19_H_16_N_2_O_6_S, yellow solid, yield: 51.5% FT-IR (cm^−1^): 3485 (OH), 2943 (C-H), 2620 (CH), 1867 (C=O), 1673 (C=C); ^1^HNMR (400 MHz, DMSO-d_6_, *δ*, ppm): 12.34 (s, O=C-NH, 1-H), 10.10 (s, ar-OH, 1-H), 9.45 (s, ar-OH, 1-H), 7.43 (s, CH, 1-H), 7.14–7.13 (d, ar-H, 2H), 7.02–7.00 (m, ar-H, 3-H), 6.89–6.87 (d, ar-H, 1-H), 4.84 (s, CH_2_, 2-H), 3.83 (s, CH3, 3-H); ^13^C-NMR (100 MHz, DMSO-d_6_, *δ*, ppm): 176.8, 172.8, 158.8, 156.3, 149.8, 148.2, 137.1, 135.0, 134.8, 130.9, 129.2, 128.5, 128.5, 124.0, 123.3, 119.6, 118.4, 63.3 and 55.5; the LC-MS (*m*/*z*) value for C_19_H_16_N_2_O_6_S is 400.41, found to be 400.61 (M^+^) (Figures S5–S8).

**(*E*)-2-(5-(4hydroxy-3-methoxybenzylidene)-2,4-dioxothiazolidin-3-yl)-N-(2-hydroxyphenyl)acetamide (**D3**):**

Molecular formula: C_19_H_16_N_2_O_6_S, yellow solid, yield: 52.3%, FT-IR (cm^−1^): 3434 (NH), 2941 (C=CH), 2762 (CH), 1658 (C=C); ^1^H-NMR (400 MHz, DMSO-d_6_, *δ*, ppm): 11.88 (s, ar-OH, 1-H), 10.34 (s, O=C-NH, 1-H), 9.43 (s, ar-OH, 1H), 7.11–7.10 (d, ar-H, 1-H), 6.98–6.95 (m, ar-H, 1H), 6.84–6.82 (d, ar-H, 1-H), 4.61 (s, CH2, 2H), 3.79 (s, CH3, 3-H); ^13^CNMR (100 MHz, DMSO-d_6_, *δ*, ppm): 182.7, 175.8, 158.8, 156.3, 149.8, 148.2, 137.1, 135.0, 134.8, 130.9, 129.2, 128.5, 128.5, 124.0, 123.3, 119.6, 118.4, 55.4 and 40.1; the LC-MS (*m*/*z*) value for C_19_H_16_N_2_O_6_S is 400.41, found to be 400.30 (M^+^) (Figures S9–S12).

**(*E*)-2-(5-(4hydroxy-3-methoxybenzylidene)-2,4-dioxothiazolidin-3-yl)-N-(4-nitrophenyl)acetamide (**D4**):**

Molecular formula: C_19_H_15_N_3_O_7_S, yellow solid, yield: 22.4%, FT-IR (cm^−1^): 3431 (NH), 3064 (C=C-H), 2942 (-CH), 2761 (CH), 1668 (C=C), 1550 (NO2), 810 (C-NO2); ^1^H-NMR (400 MHz, DMSO-d_6_, *δ*, ppm): 11.14 (s, O=C-NH, 1H), 10.01 (s, ar-OH, 1-H), 9.45 (s, CH, 1-H), 8.26–8.24 (d, Ar-H, 1-H), 7.92–7.84 (d, Ar-H, 1-H), 7.37 (s, ar-H, 1-H), 7.14–7.13 (d, ar-H, 1-H), 4.39 (s, CH2, 2-H), 3.82 (s, CH3, 3-H); ^13^CNMR (100 MHz, DMSO-d_6_, *δ*, ppm): 173.7, 167.3, 165.4, 165.1, 144.6, 142.5, 134.6, 128.2, 126.3, 126.2, 125.1, 124.4, 124.1, 123.1, 119.0, 116.3, 116.3, 55.5 and 44.1; the LC-MS (*m*/*z*): valued for C_19_H_15_N_3_O_7_S is 429.40, found to be 429.33 (M^+^) (Figures S13–S16).

**(*E*)-*N*-(4-fluorophenyl)-2-(5-(4hydroxy-3-methoxybenzylidene)-2,4-dioxothiazolidin-3-yl)acetamide (**D5**):**

Molecular formula: C_19_H_15_FN_2_O_5_S, yellow solid, yield: 61.09% FT-IR (cm^−1^): 3489 (OH), 3274 (NH), 3223 (C=C-H), 3164 (Ar, CH), 3100 (AR, CH), 1668 (C=C), 1593 (C=N), 888 (C-F); ^1^HNMR (400 MHz, DMSO-d_6_, *δ*, ppm):10.46 (s, O=C-NH, 1H), 9.45 (s, Ar-OH, 1-H), 7.64–7.60 (m, Ar-H, 1-H), 7.25 (s, ar-H, 1-H), 4.26 (s, CH_2_, 2-H), 3.85 (s, CH_3_, 3-H); ^13^CNMR (100 MHz, DMSO-d_6_, *δ*, ppm): 182.4, 175.7, 163.9, 147.6, 146.9, 134.4, 131.8, 127.1, 123.5, 122.6, 120.9, 116.3, 115.7, 115.6, 115.3, 114.4, 112.9, 55.6 and 48.9; the LC-MS (*m*/*z*): valued for C_19_H_15_FN_2_O_5_S is 402.4, found to be: 402.4 (M^+^) (Figures S17–S20).

### 3.6. Biological Evaluations

**MTT Assay Cytotoxicity Analysis:**

The lethal concentration of synthetic chemicals (**D1**–**D5**) was evaluated using the MTT test. Using various dosages of the generated chemicals, a standard MTT experiment was performed against L6 cells at 570 nanometres in a 96-well microplate reader. It used a 1% antibiotic–antimycotic solution. The growth medium was made up of DMEM supplemented with 10% FBS which supplied the necessary growth factors and nutrients needed for the maintenance of cells. To separate the cells, we used 0.25% trypsin-EDTA after growing to about 70% confluence, and they were subsequently subcultured. Cell cultures were maintained at 37 °C in a humidified incubator with 5% CO_2_. Each experiment used cells from three passages. At concentrations as high as 250 μM, the thiazolidinedione derivatives **D1**–**D5** showed no toxicity and high compatibility with L6 myoblast cells [37].

### 3.7. α-Amylase In Vitro Inhibitory Assay 

After adding 20 µL of a 0.5 mg/mL, 200 microliters of 0.02 M phosphate buffer were used to prepare the α-amylase solution at a pH of 6.9; the mixture was then allowed to stay at room-temperature conditions for around 10 min. Then, test samples with concentrations from 10 to 50 µg/mL were added, each volume being 250 µL. After adding 200 microliters of 1% starch, the mixture incubated for another ten minutes at 25 °C. The procedure was stopped by including 400 µL of DNS reagent and heating the mixture for five minutes at 70 °C. Measurements were taken with a microplate reader, absorbance at 540 nm. Acarbose is the positive control; each experiment was conducted three times. The control reaction mixture was prepared without the test sample [38]. 

The formula below was used to calculate the α-amylase inhibition:

$$\begin{array}{l} \%\mathrm{Inhibition}=[(C-T)/C]\times 100\\ C\text{—}\mathrm{absorbance}\;\mathrm{of}\;\mathrm{control},\;T\text{—}\mathrm{absorbance}\;\mathrm{of}\;\mathrm{sample}. \end{array}$$

### 3.8. α-Glucosidase In Vitro Inhibitory Assay 

A reaction mixture was prepared using the enzyme α-glucosidase, of ten microliters, 0.1 molar phosphate buffer (pH-6.8) of 50 microliters, and test samples of 20 microliters at various concentrations between 10 and 50 µg/mL. This reactant mixture was then added into a 96-well plate and kept for incubation at 37 °C for a period of 15 min. After this, twenty microliters of p-nitrophenyl D-glucopyranoside solution were added as a substrate. The mixture was again incubated at 37 °C for an additional 20 min. The reaction stopped when 50 µL of 0.1 M Na_2_CO_3_ was added. Absorbance measurement was done at 405 nm using a microplate reader. Acarbose is the positive control, and the control was a reaction mixture where the sample for testing is missing. We conducted three runs of each experiment [39].

The formula below was used to calculate the α-glucosidase inhibition:

$$\begin{array}{l} \%\mathrm{Inhibition}=[(C-T)/C]\times 100\\ C\text{—}\mathrm{absorbance}\;\mathrm{of}\;\mathrm{control},\;T\text{—}\mathrm{absorbance}\;\mathrm{of}\;\mathrm{sample}. \end{array}$$

### 3.9. Design of the Experiment and Drosophila Culture

The Oregon-R flies used in this study were acquired from the Drosophila Lab at the Nitte University in Mangalore, India. They were kept using a medium made of maize and agar and using a 12 h light and dark cycle and relative humidity of about 65 to 70 percent. To study the toxicity, we used *Drosophila melanogaster* larvae at different concentrations of the drug, from 500 ppm to 2000 ppm, to find the LC50 values for compounds **D1**, **D4**, and **D5**. We calculated the LC50 values based on the mortality rates of the Drosophila larvae. We chose sub-lethal concentrations, specifically ≤12,000 ppm, to examine their effect on lowering glucose levels. Male and female pupae were separated for ten days. They were fed a high-sugar diet and various food types that included a high-sugar diet at consistent amounts of 300 PPM and 600 PPM of chemicals **D1** and **D5**. To prepare the samples, an ice-cold phosphate-buffered saline of volume 0.2 mL was used to homogenize twenty flies at a pH of 7.4. After homogenization, the samples were spun in a centrifuge at 10,000 rpm for 15 min at 4 °C. The glucose content of each fly homogenate was measured following the guidelines from Agape Diagnostics Ltd. in India. The control group went through the same conditions as the other groups during the evaluation. The only difference was the absence of HSD and chemicals. Three runs of each experiment were done. The control group’s results were shown as percentages [40].

## 4. Conclusions

This research successfully developed a new series of 2,4-Thiazolidinedione (TZD) derivatives and evaluated their effectiveness as antihyperglycemic agents using both computer modeling and laboratory research. These results also show that these compounds may serve as the basis for more effective medications for the management of metabolic disorders. In silico studies showed that the synthesized molecules have desirable drug-like and pharmacokinetic properties, and that they have low potential for toxicity. The molecular docking analysis and MM-GBSA testing demonstrated that lead compounds, **D1** and **D5**, have strong and stable interactions with their target protein, 3TOP. The results of the in vitro tests demonstrated that **D1** and **D5** inhibit the activity of carbohydrate-hydrolyzing enzymes, further validating their mechanisms of action. Further testing in *Drosophila melanogaster* demonstrated a dose-dependent lowering of glucose levels when **D1** and **D5** were administered to hyperglycemic animals. In comparison to the standard drug, Acarbose, **D1** and **D5** both demonstrated a better quality of efficacy, pharmacokinetic properties, and therapeutic safety. **D1** and **D5** are therefore good lead candidates for the development of safe and effective new medications to treat diabetes. Future work is needed, including more formalized toxicology testing and clinical trials, to support the therapeutic potential of **D1** and **D5** in treating diabetes.

## Figures and Tables

**Table 1:** Assessment of Lead Compounds (**D1**–**D5**) Based on Lipinski’s rule of five and drug-likeness scores.

Comp	Molecular Weight	Log *p* (QlogPo/w)	Donor HB	Acceptor HB	Rule of Five
Acceptable Range	≤500	<5	≤5	≤10	<5
**D1**	400.405	2.092	3	7.75	0
**D2**	400.405	2.162	3	7.75	0
**D3**	400.405	2.286	3	7.75	0
**D4**	429.403	2.184	2	8	0
**D5**	402.396	3.161	2	7	0
**Acarbose**	645.6109	−7.044	13	31.39	3

Estimated octanol–water partition coefficient; **Donor HB:** number of hydrogen bond donors; **Acceptor HB:** number of hydrogen bond acceptors.

**Table 2:** Predicted ADME parameters of the lead compounds (**D1**–**D5**) using QikProp.

Comp	%HOA	QPlogS	QPPCaco	QPlogBB	QppMDCK	QplogKHSA	#Metab	QPlogKp
Acceptable Range	<25% Low >80% High	−6.5–−0.5	<25 Poor; >500 Great	−3.0–1.2	<25 Poor; >500 Great	−1.5–1.5	(1–8)	−8.0–−1.0
**D1**	73.94	−4.2	87.3	−1.9	64.09	−0.08	4	−3.81
**D2**	74.79	−4.3	92.50	−1.9	68.15	−0.07	5	−3.74
**D3**	77.26	−4.6	115.7	−1.9	86.83	−0.06	5	−3.50
**D4**	67.55	−4.7	35.8	−2.4	24.48	0.02	4	4.60
**D5**	88.95	−5.4	269.4	−1.4	390.3	0.12	3	−2.90
Acarbose	0	1.34	0.03	−6.1	0.007	−2.72	14	−10.5

HOA: percent human oral absorption; QPlogS: estimated aqueous solubility; QPPCaco: predicted Caco-2 cell membrane permeability nm/s; QPlogBB: predicted brain–blood partition coefficient; QppMDCK: estimated MDCK cell permeability; QplogKHSA: predicted human serum albumin binding affinity; #metab: estimated count of metabolic transformations; QPlogKp: predicted skin permeability coefficient.

**Table 3:** In silico toxicity prediction and drug-likeness scoring of synthetic derivatives (**D1**–**D6**).

Comp	Drug Relevant Properties				Toxicity			
	Clog *p*	Solubility	Drug Likeness	Drug Score	Tumgn	RepE	IrE	Mutgn
**D1**	1.77	−3.49	6.25	0.79				
**D2**	1.77	−3.49	5.92	0.79				
**D3**	1.77	−3.49	6.03	0.79				
**D4**	1.2	−4.24	−9.26	0.35				
**D5**	2.22	−4.1	4.82	0.73				
Acarbose	−7.91	0.67	−9.31	0.29				

Tumgn: tumorigenic (risk of causing tumors/cancer); RepE: reproductive effective (risk of affecting fertility/reproductive system); IrE: irritant effect (risk of causing skin/eye irritation); Mutgn: mutagenic (risk of causing genetic mutations/DNA damage); green color: indicates drug-confrom behavior.

**Table 4:** Molecular docking results of 2,4-thiazolidinedione compounds (**D1**, **D4** and **D5**).

Code	Binding Energy (kcal/mol)	H-Bond Residues	No. of H-Bond	Other Residues
**D1**	−7.74033	*Gln1372*, *Trp1369*, *Hie1584*	3	Arg1377, Gly1365, Ile1587, Thr1586, Phe1560, Phe1599, Arg1510, Arg1582, Asp1526, Gly1525, Asp1555, Trp1523, Trp1418, Asp1420, Met1421, Ile1280, Asp1279, Ile1315, Trp1355, Tyr1251
**D4**	−7.34624	*Trp1369*, *Arg1377*, *Asp1279*, *Hie1584*	5	Asp1157, Asp1526, Gly1525, Trp1523, Arg1582, Met1421, Trp1418, Phe1560, Phe1559, Trp1355, Asp1555, Arg1510, Ile1315, Thr1586, Ile1280, Ile1587, Tyr1251, Val1363, Gly1365, Gln1372
**D5**	−7.39806	*Trp1369*, *Asp1279*, *Hie1584*	3	Asp1157, Phe1560, Phe1559, Trp1523, Gly1525, Asp1526, Trp1355, Asp1555, Met1421, Trp1418, Arg1510, Tyr1251, Ile1315, Arg1582, Ile1280, Thr1586, Ile1587, Arg1377, Gln1372
(Std) Acarbose	−10.739	*Lys1164*, *Gln1158*, *Asp1526*, *Asp1420*, *Asp1279*, *Asp1157*	9	Trp1369, Phe1560, Phe1559, Trp1523, Trp1355, Met1421, Trp1418, Thr1586, Arg1510, Hie1584, Ile1280, Ile1315, Tyr1251, Ser1425, Phe1427, Lys1460, Pro1159

**Table 5:** Binding free energy calculation using MM-GBSA approach.

Bioactive	ΔGbind (Kcal/mol)	ΔGbind Coulomb	ΔGbind Covalent	ΔGbind Vander	ΔGbind H-Bond	ΔGbind Lipophilic
**D1**	−40.9544	−29.4358	10.5409	−48.622	−3.2872	−24.0058
**D2**	−35.3254	−24.2975	8.3381	−49.507	−3.3536	−21.8795
**D3**	−34.0034	−20.9655	8.3134	−48.120	−2.1366	−22.0775
**D4**	−35.2175	−17.5731	12.1111	−51.068	−2.7761	−24.4121
**D5**	−35.1563	−19.6545	6.8069	−47.497	−2.1556	−24.2533
**Acarbose**	1.0053	−50.3553	19.3717	−35.251	−8.6106	−27.3524

**ΔG bind:** total free energy of binding; **ΔG bind Coulomb:** coulombic (electrostatic) interaction energy; **ΔG bind Covalent:** covalent or internal bonding energy; **ΔG bind (VdW):** van der Waals interaction energy; **ΔG bind (H-bond):** hydrogen-bonding contribution; **ΔG bind (Lipo):** lipophilic energy (representing hydrophobic interactions based on solvent-accessible surface area).

**Table 6:** In vitro antidiabetic activity of the synthesized compounds (**D1**–**D5**).

Compound	IC_50_ Values	
	α-Amylase (μM)	α-Glucosidase (μM)
**D1’**	33.86 ± 2.1	29.81 ± 3.2
**D2**	44.16 ± 0.8	36.99 ± 0.8
**D3**	73.32 ± 1.4	37.99 ± 1.4
**D4**	52.31 ± 1.56	36.51 ± 2.4
**D5**	37.55 ± 1.7	32.43 ± 1.2
**Acarbose (Std)**	24.35 ± 1.44	23.73 ± 1.22

All the results expressed are mean of three individual replicates (n = 3, ±SD).

## Data Availability

The data supporting the findings of this study are available from the corresponding author upon reasonable request.

## Supplementary Materials

The following supporting information can be downloaded at https://www.mdpi.com/article/10.3390/molecules31183160/s1, Spectral characterization.
