# Supplementary material for: Synthesis and Antidiabetic Evaluation of Novel 2,4-Thiazolidinedione Derivatives Targeting Key Carbohydrate-Digesting Enzymes
Source: Molecules. 2026 Sep 8;31(18):3160. doi: 10.3390/molecules31183160 (PMC13609656; doi:10.3390/molecules31183160)
Supplement: Supplementary file 1 [file molecules-31-03160-s001.zip › molecules-4446293-supplementary.pdf]

# Spectral Characterization

**D1: (E)-2-(5-(4-hydroxy-3-methoxybenzylidene)-2,4-dioxothiazolidin-3-yl)-N-(4-hydroxyphenyl)acetamide**

Molecular formula: $C_{19}H_{16}N_2O_6S$, yellow solid, yield:48.5%, FT-IR ($cm^{-1}$): 3433 (N-H), 2762 (C-H), 1659 (C=C), 1592 (C=O); $^1$H-NMR (400MHz, DMSO-d6, $\delta$, ppm): 12.34(O=C-NH-), 10.01(-OH), 9.45(-OH), 7.43(ar-H), 7.14-7.13(d, ar-H, 2H), 7.02-7.00(m), 6.89-6.87(d, ar-H), 4.83(s, $CH_2$), 3.83(s, $CH_3$); $^{13}$C-NMR (100 MHz, DMSO-$d_6$, $\delta$, ppm): 176.8, 172.8, 158.8, 156.3, 149.8, 148.2, 137.1, 135.0, 134.8, 130.9, 129.2, 128.5, 128.5, 124.0, 123.3, 119.7, 118.6, 63.4 and 55.5; The LC-MS (m/z) value for $C_{19}H_{16}N_2O_6S$ is 400.41; found to be 400.50 (M+).

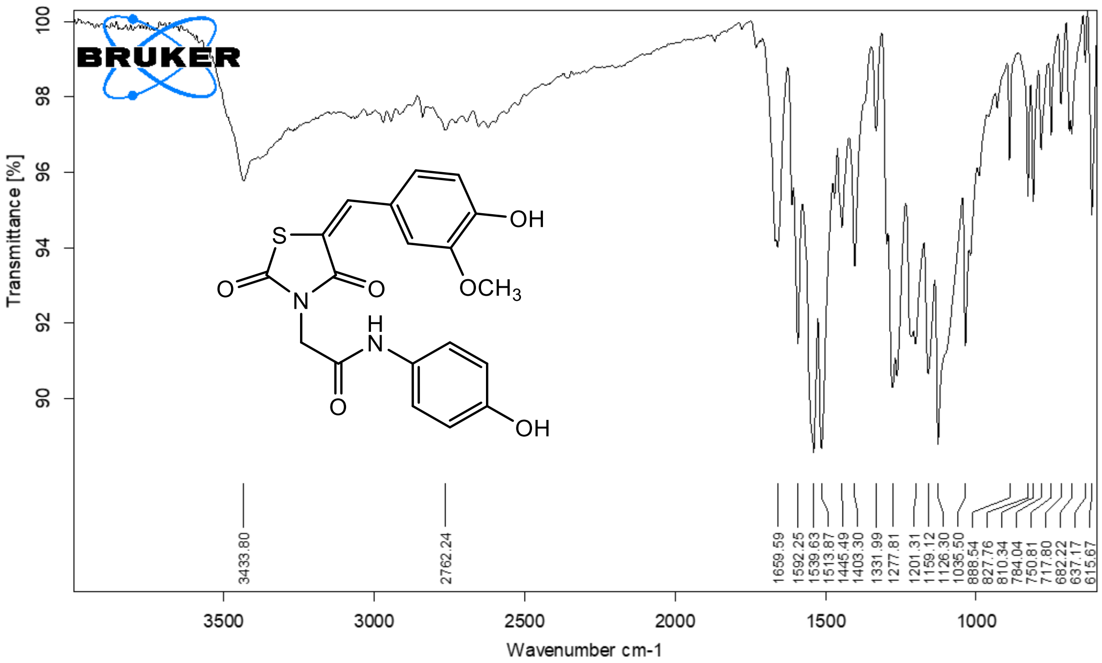


**Figure S1: IR spectrum of Compound D1: (E)-2-(5-(4-hydroxy-3-methoxybenzylidene)-2,4-dioxothiazolidin-3-yl)-N-(4-hydroxyphenyl)acetamide**

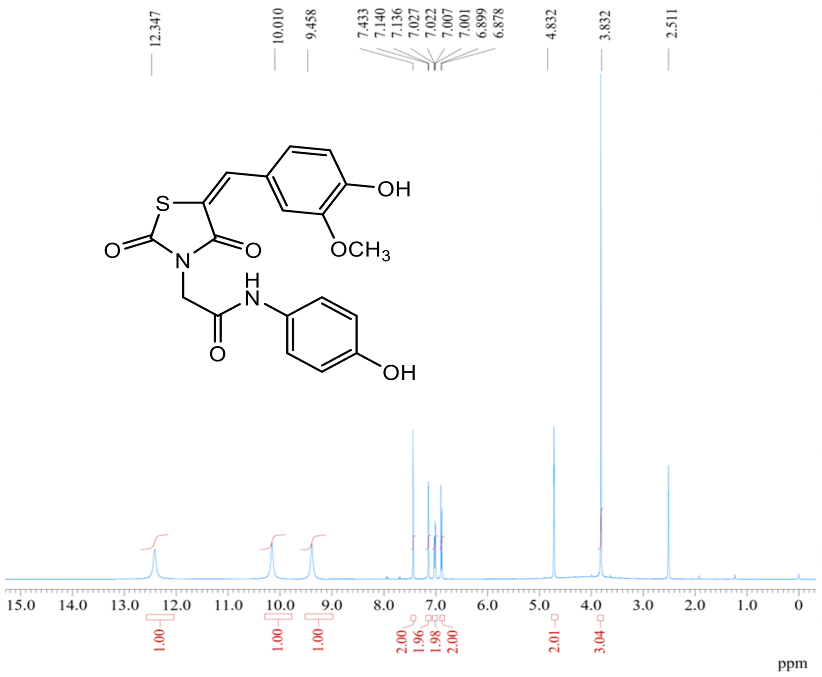


**Figure S2: 1H NMR spectrum of Compound D1: (E)-2-(5-(4-hydroxy-3-methoxybenzylidene)-2,4-dioxothiazolidin-3-yl)-N-(4-hydroxyphenyl)acetamide**

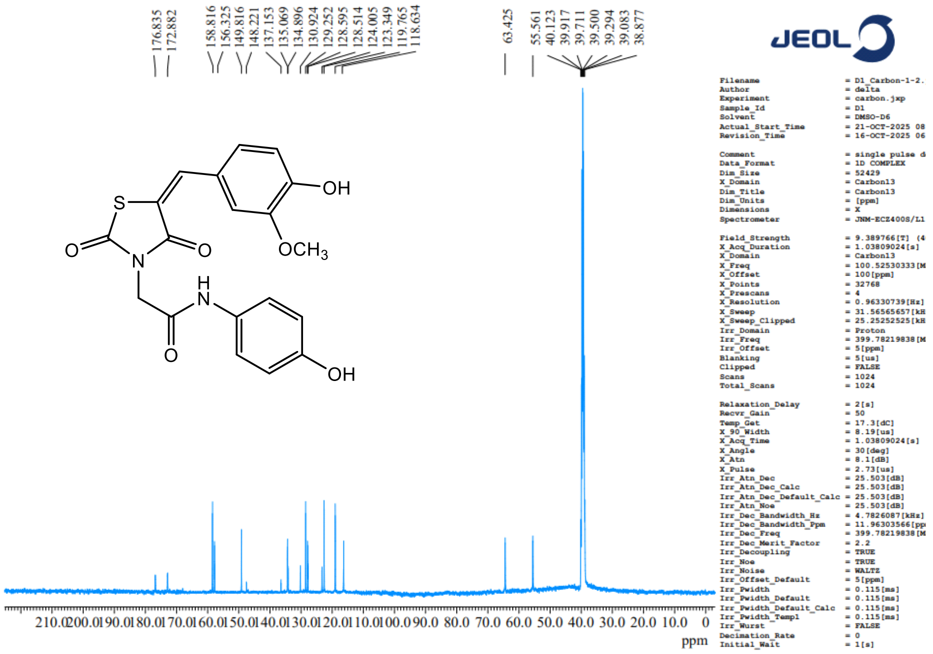


**Figure S3: 13C NMR spectrum of Compound D1: (E)-2-(5-(4-hydroxy-3-methoxybenzylidene)-2,4-dioxothiazolidin-3-yl)-N-(4-hydroxyphenyl)acetamide**

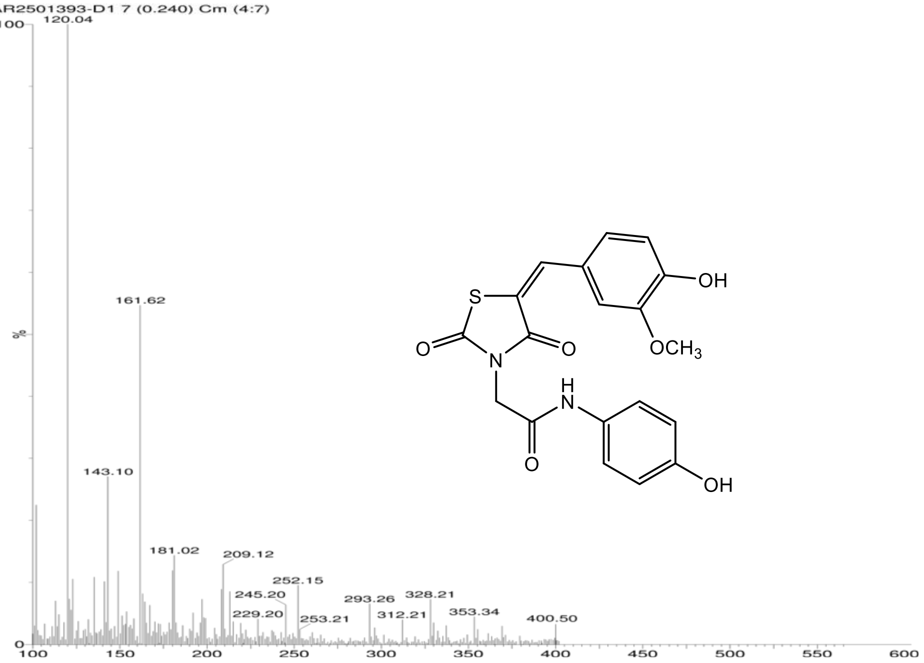


**Figure S4: Mass spectrum of Compound D1: (E)-2-(5-(4-hydroxy-3-methoxybenzylidene)-2,4-dioxothiazolidin-3-yl)-N-(4-hydroxyphenyl)acetamide**

**D2: (E)-2-(5-(4-hydroxy-3-methoxybenzylidene)-2,4-dioxothiazolidin-3-yl)-N-(3-hydroxyphenyl)acetamide**

Molecular formula: $C_{19}H_{16}N_2O_6S$, yellow solid, yield: 51.5% FT-IR ($cm^{-1}$): 3485(OH), 2943(C-H), 2620(CH), 1867(C=O), 1673(C=C); $^1$HNMR (400MHz, DMSO-d6, $\delta$, ppm):12.34 (s, O=C-NH, 1-H), 10.10(s, ar-OH, 1-H), 9.45(s, ar-OH, 1-H), 7.43(s, CH, 1-H), 7.14-7.13(d, ar-H, 2H), 7.02-7.00(m, ar-H, 3-H), 6.89-6.87(d, ar-H, 1-H), 4.84(s, $CH_2$, 2-H), 3.83(s, CH3, 3-H); $^{13}$C-NMR (100 MHz, DMSO-$d_6$, $\delta$, ppm): 176.8, 172.8, 158.8, 156.3, 149.8, 148.2, 137.1, 135.0, 134.8, 130.9, 129.2, 128.5, 128.5, 124.0, 123.3, 119.6, 118.4, 63.3 and 55.5; The LC-MS (m/z) value for $C_{19}H_{16}N_2O_6S$ is 400.41; found to be 400.61 (M+).

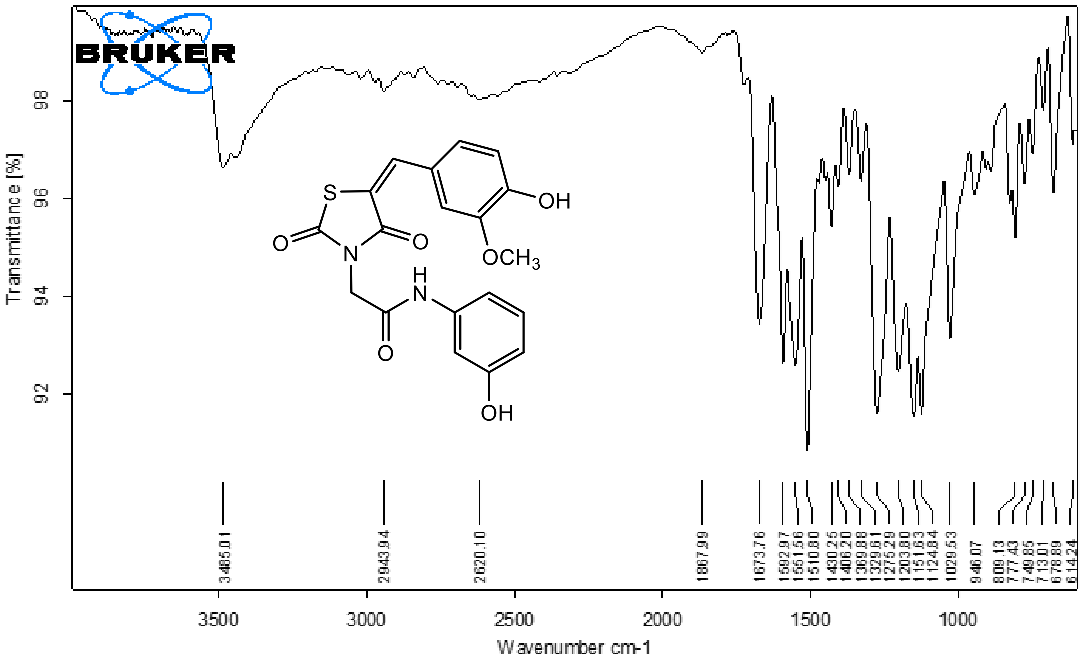


**Figure S5: IR spectrum of Compound D2: (E)-2-(5-(4-hydroxy-3-methoxybenzylidene)-2,4-dioxothiazolidin-3-yl)-N-(3-hydroxyphenyl)acetamide**

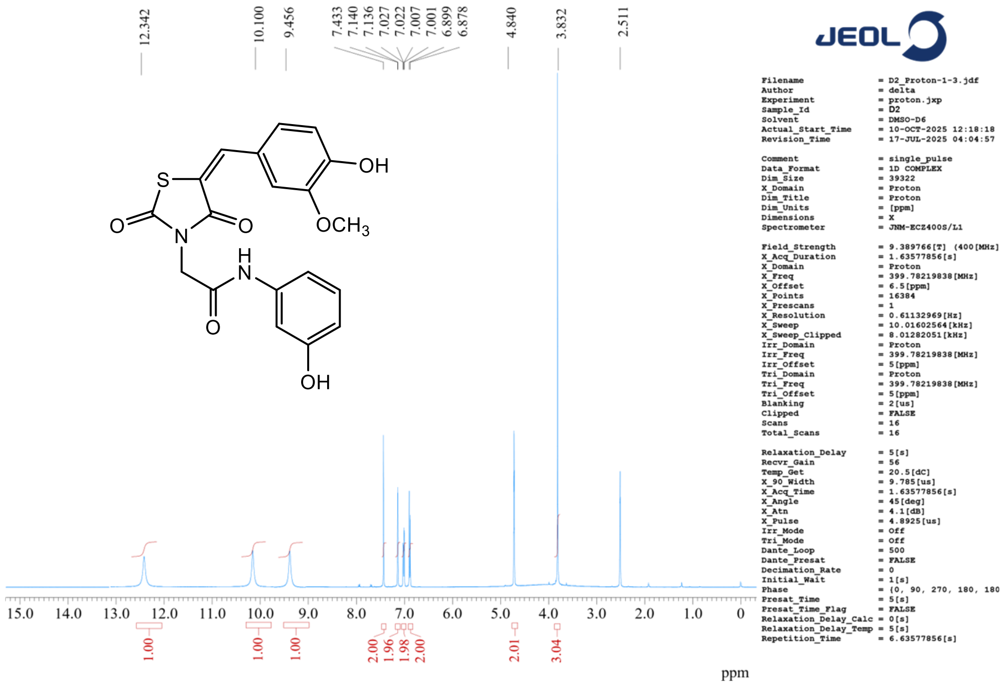


**Figure S6: 1H NMR spectrum of Compound D2: (E)-2-(5-(4-hydroxy-3-methoxybenzylidene)-2,4-dioxothiazolidin-3-yl)-N-(3-hydroxyphenyl)acetamide**

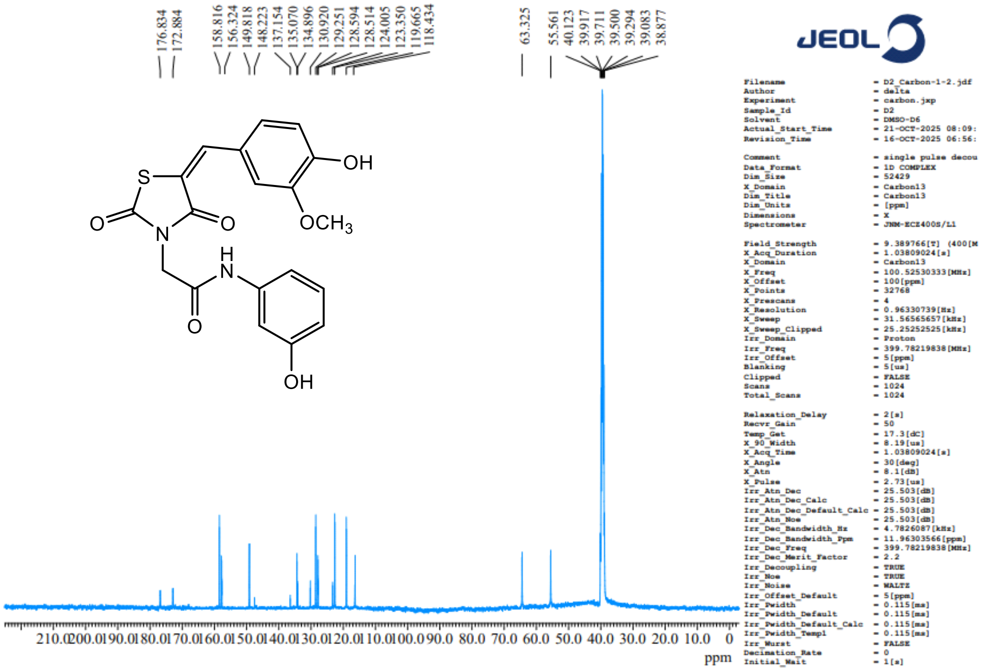


**Figure S7: 13C NMR spectrum of Compound D2: (E)-2-(5-(4-hydroxy-3-methoxybenzylidene)-2,4-dioxothiazolidin-3-yl)-N-(3-hydroxyphenyl)acetamide**

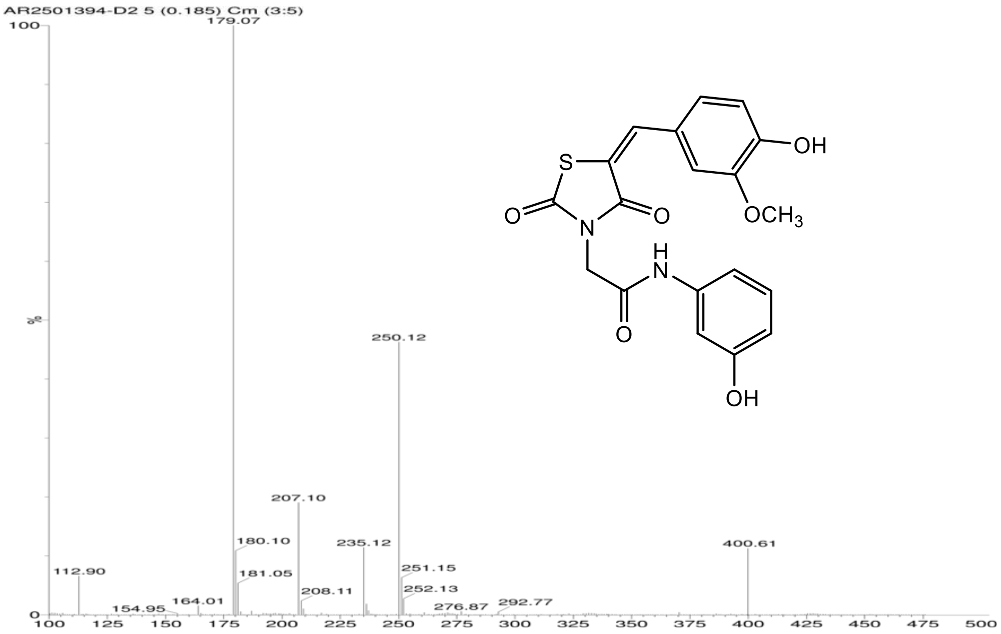


**Figure S8: Mass spectrum of Compound D2: (E)-2-(5-(4-hydroxy-3-methoxybenzylidene)-2,4-dioxothiazolidin-3-yl)-N-(3-hydroxyphenyl)acetamide**

**(E)-2-(5-(4-hydroxy-3-methoxybenzylidene)-2,4-dioxothiazolidin-3-yl)-N-(2-hydroxyphenyl)acetamide**

Molecular formula: $C_{19}H_{16}N_2O_6S$, yellow solid, yield: 52.3%, FT-IR ($cm^{-1}$): 3434(NH), 2941(C=CH), 2762(CH), 1658(C=C); $^1$H-NMR (400MHz, DMSO-d6, $\delta$, ppm): 11.88(s, ar-OH, 1-H), 10.34(s, O=C-NH, 1-H), 9.43(s, ar-OH, 1H), 7.11-7.10(d, ar-H, 1-H), 6.98-6.95(m, ar-H, 1H), 6.84-6.82(d, ar-H, 1-H), 4.61(s, CH2, 2H), 3.79(s, CH3, 3-H); $^{13}$CNMR (100 MHz, DMSO-$d_6$, $\delta$, ppm):182.7, 175.8, 158.8, 156.3,149.8, 148.2, 137.1, 135.0, 134.8, 130.9, 129.2, 128.5, 128.5,124.0, 123.3, 119.6, 118.4, 55.4 and 40.1; The LC-MS (m/z) value for $C_{19}H_{16}N_2O_6S$ is 400.41; found to be 400.30 (M+).

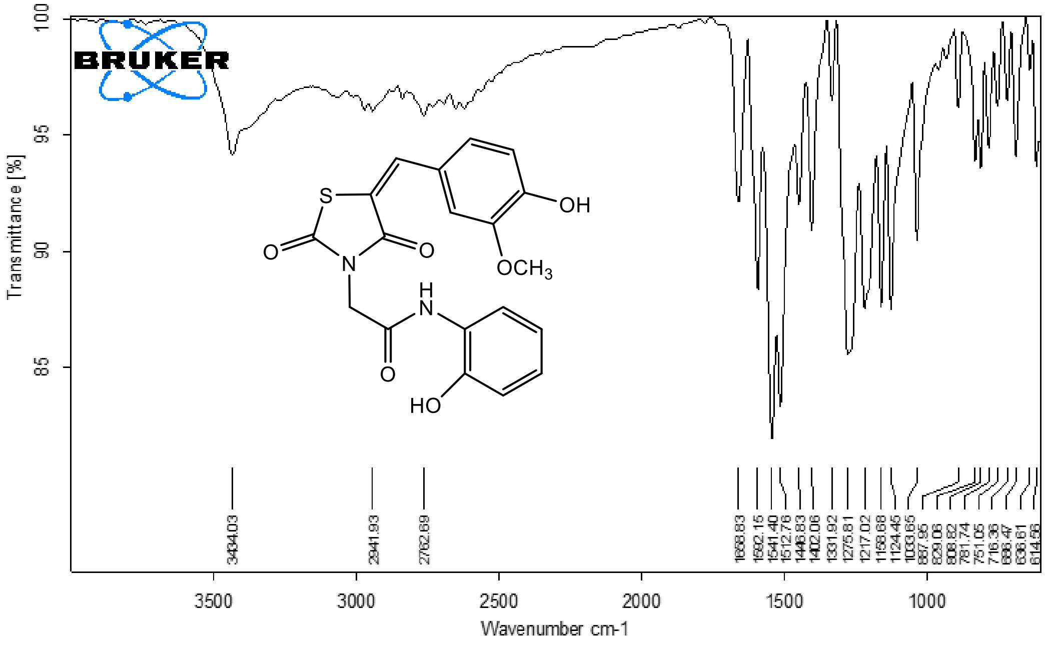


**Figure S9: IR spectrum of Compound D3: (E)-2-(5-(4-hydroxy-3-methoxybenzylidene)-2,4-dioxothiazolidin-3-yl)-N-(2-hydroxyphenyl)acetamide**

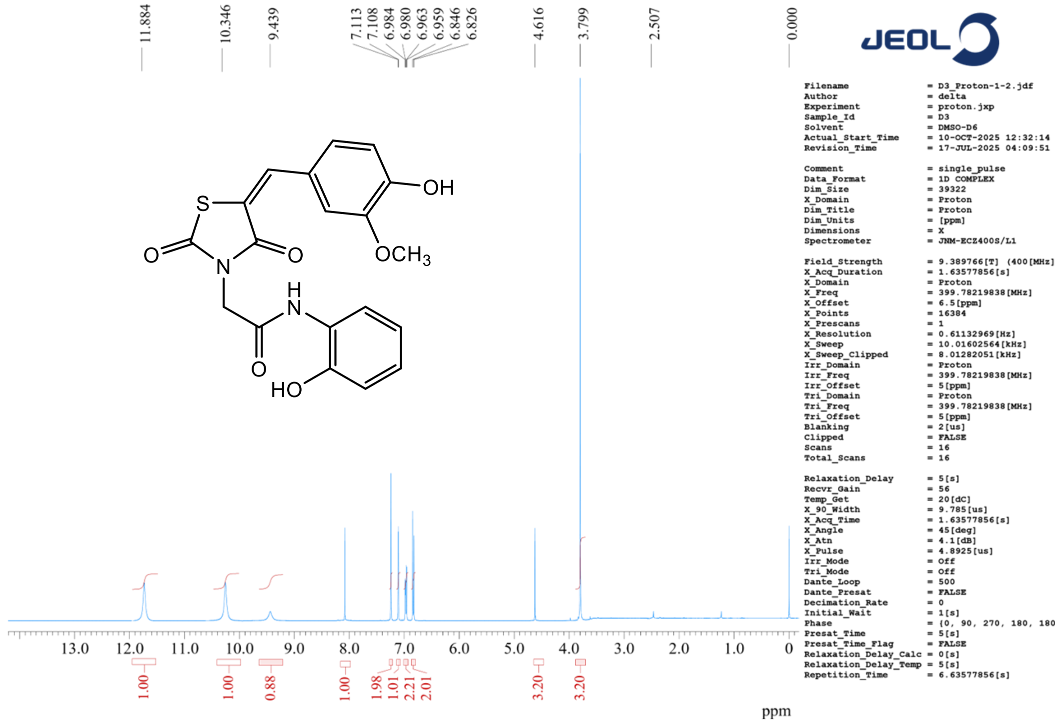


**Figure S10: 1H NMR spectrum of Compound D3: (E)-2-(5-(4-hydroxy-3-methoxybenzylidene)-2,4-dioxothiazolidin-3-yl)-N-(2-hydroxyphenyl)acetamide**

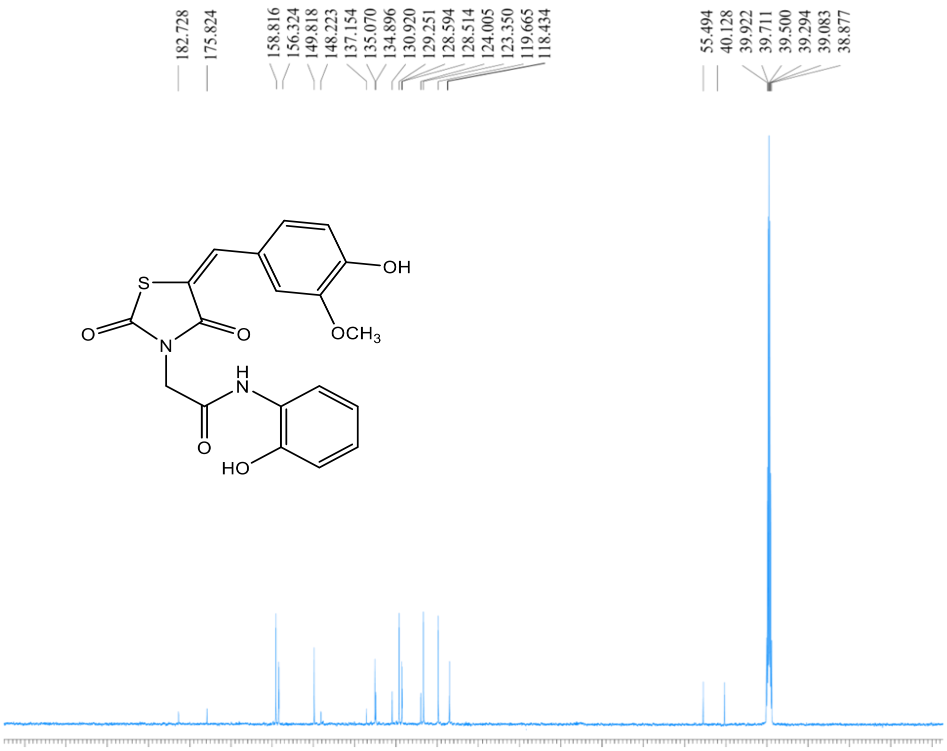


**Figure S11: 13C NMR spectrum of Compound D3: (E)-2-(5-(4-hydroxy-3-methoxybenzylidene)-2,4-dioxothiazolidin-3-yl)-N-(2-hydroxyphenyl)acetamide**

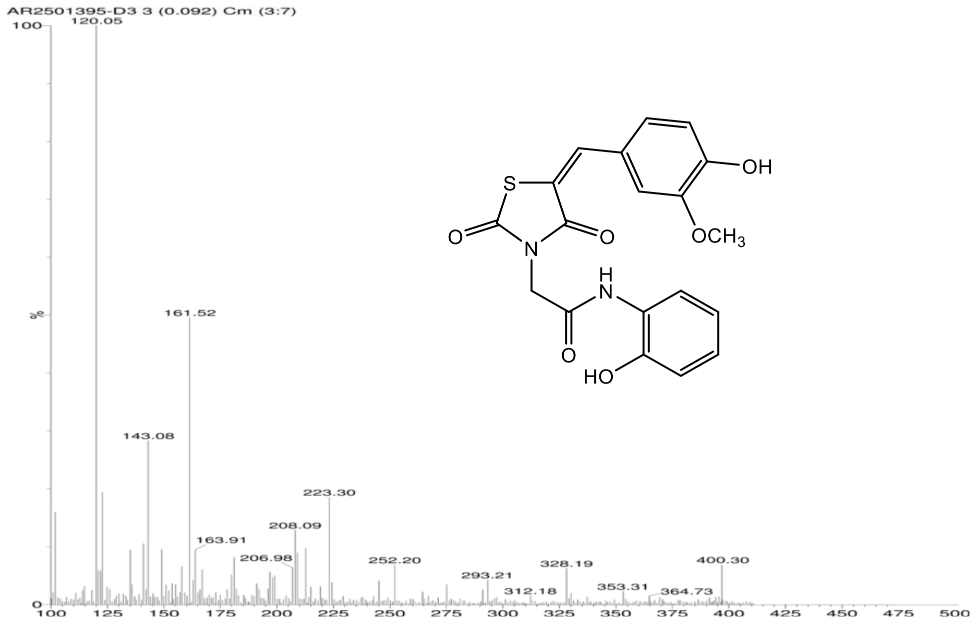


**Figure S12: Mass spectrum of Compound D3: (E)-2-(5-(4-hydroxy-3-methoxybenzylidene)-2,4-dioxothiazolidin-3-yl)-N-(2-hydroxyphenyl)acetamide**

**D4: (E)-2-(5-(4-hydroxy-3-methoxybenzylidene)-2,4-dioxothiazolidin-3-yl)-N-(4-nitrophenyl)acetamide**

Molecular formula: $C_{19}H_{15}N_3O_7S$, yellow solid, yield: 22.4%, FT-IR ($cm^{-1}$):3431(NH), 3064(C=C-H), 2942(-CH), 2761(CH), 1668(C=C), 1550(NO2), 810(C-NO2); $^{1}$H-NMR (400MHz, DMSO-d6, $\delta$, ppm):11.14(s, O=C-NH, 1H), 10.01(s, ar-OH, 1-H), 9.45(s, CH, 1-H), 8.26-8.24(d, Ar-H, 1-H), 7.92-7.84(d, Ar-H, 1-H), 7.37(s, ar-H, 1-H), 7.14-7.13(d, ar-H, 1-H), 4.39(s, CH2, 2-H), 3.82(s, CH3, 3-H); $^{13}$CNMR (100 MHz, DMSO-$d_6$, $\delta$, ppm): 173.7, 167.3, 165.4, 165.1, 144.6, 142.5, 134.6, 128.2, 126.3, 126.2, 125.1, 124.4, 124.1, 123.1, 119.0, 116.3, 116.3, 55.5 and 44.1; The LC-MS (m/z): valued for $C_{19}H_{15}N_3O_7S$ is 429.40; found to be 429.33 ($M^+$)

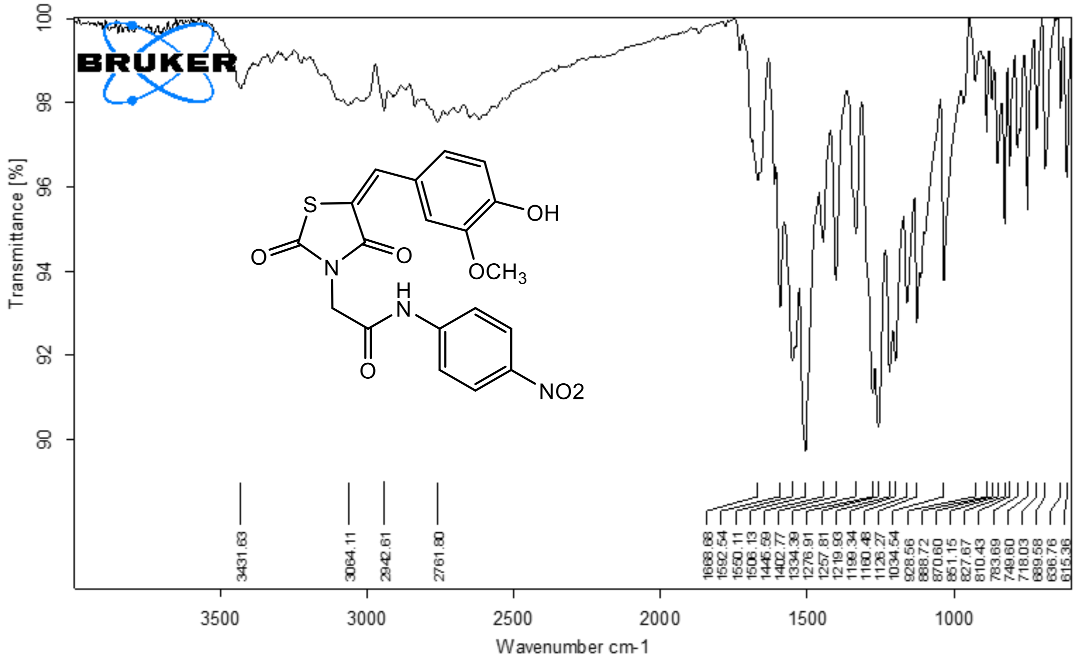


**Figure S13: IR spectrum of Compound D4: (E)-2-(5-(4-hydroxy-3-methoxybenzylidene)-2,4-dioxothiazolidin-3-yl)-N-(4-nitrophenyl)acetamide**

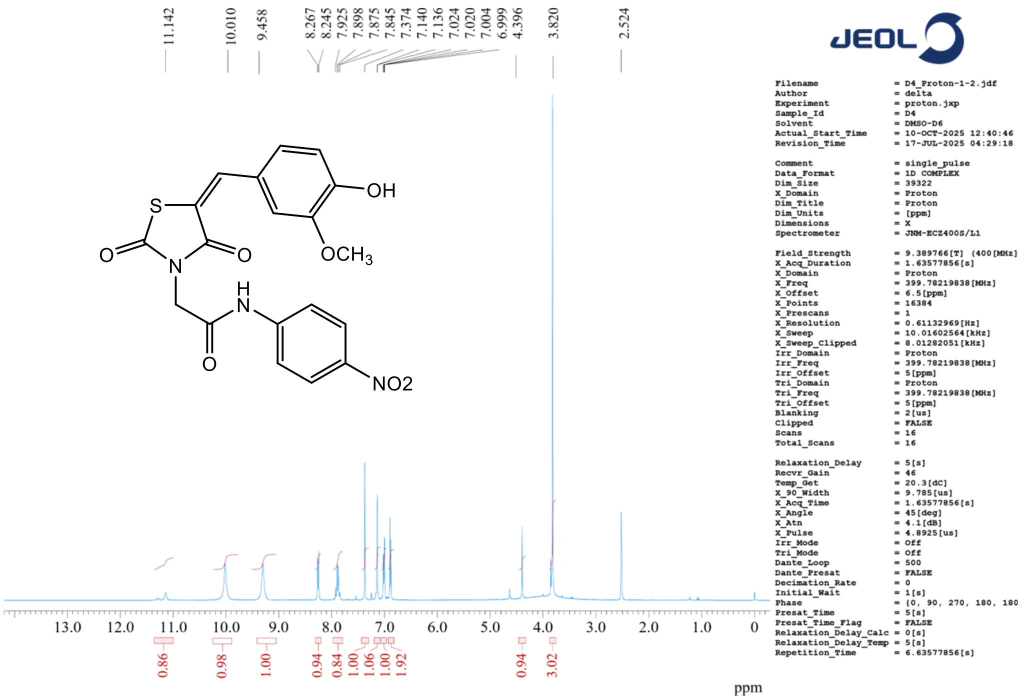


**Figure S14: 1H NMR spectrum of Compound D4: (E)-2-(5-(4-hydroxy-3-methoxybenzylidene)-2,4-dioxothiazolidin-3-yl)-N-(4-nitrophenyl)acetamide**

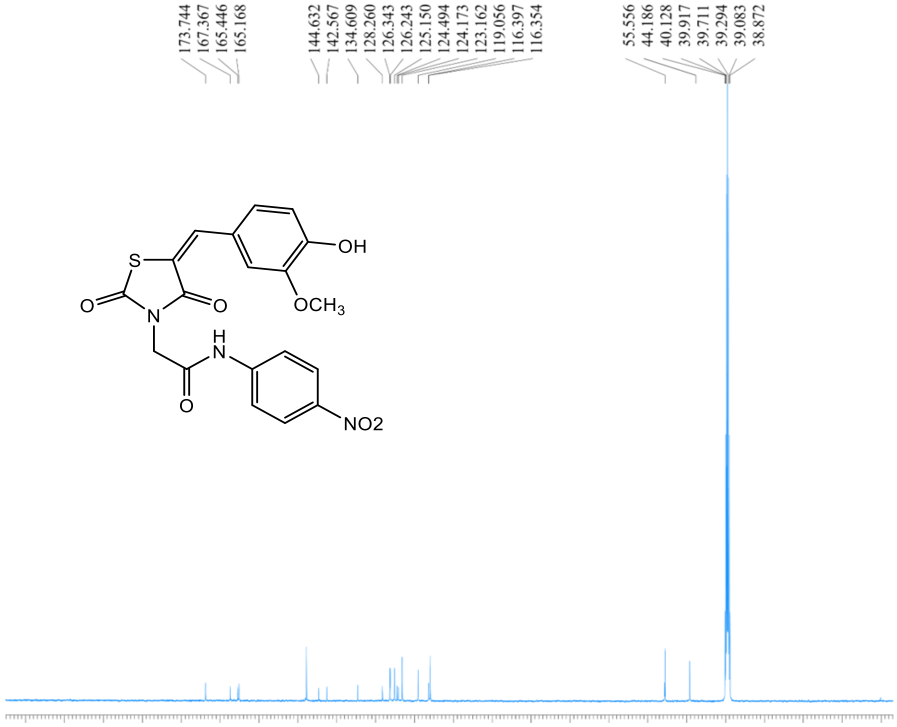


**Figure S15: 13C NMR spectrum of Compound D4: (E)-2-(5-(4-hydroxy-3-methoxybenzylidene)-2,4-dioxothiazolidin-3-yl)-N-(4-nitrophenyl)acetamide**

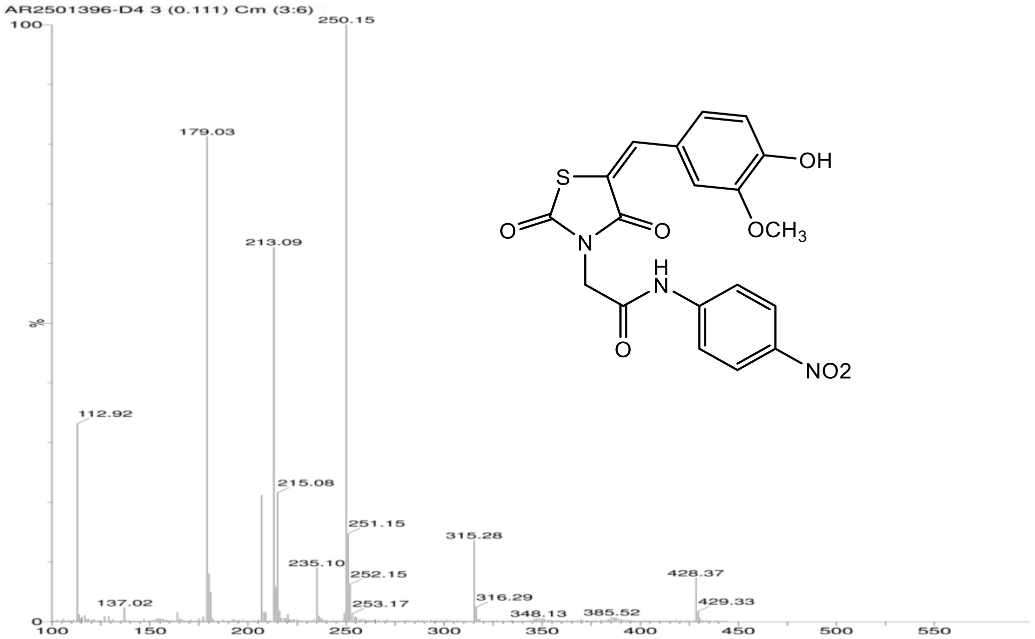


**Figure S16: Mass spectrum of Compound D4: (E)-2-(5-(4-hydroxy-3-methoxybenzylidene)-2,4-dioxothiazolidin-3-yl)-N-(4-nitrophenyl)acetamide**

**D5: (E)-N-(4-fluorophenyl)-2-(5-(4-hydroxy-3-methoxybenzylidene)-2,4-dioxothiazolidin-3-yl)acetamide**

Molecular formula: $C_{19}H_{15}FN_2O_5S$, yellow solid, yield: 61.09% FT-IR ($cm^{-1}$):3489(OH), 3274(NH), 3223(C=C-H), 3164(Ar,CH), 3100(AR, CH), 1668(C=C), 1593(C=N), 888(C-F); $^1$HNMR (400MHz, DMSO-d6, $\delta$, ppm):10.46(s, O=C-NH, 1H), 9.45(s, Ar-OH, 1-H), 7.64-7.60(m, Ar-H, 1-H), 7.25(s, ar-H, 1-H), 4.26(s, $CH_2$, 2-H), 3.85(s, $CH_3$, 3-H); $^{13}$CNMR (100 MHz, DMSO-$d_6$, $\delta$, ppm): 182.4, 175.7, 163.9, 147.6, 146.9, 134.4, 131.8, 127.1, 123.5, 122.6, 120.9, 116.3, 115.7, 115.6, 115.3, 114.4, 112.9, 55.6 and 48.9; The LC-MS (m/z): valued for $C_{19}H_{15}FN_2O_5S$ is 402.4; found to be: 402.4 ($M^+$).

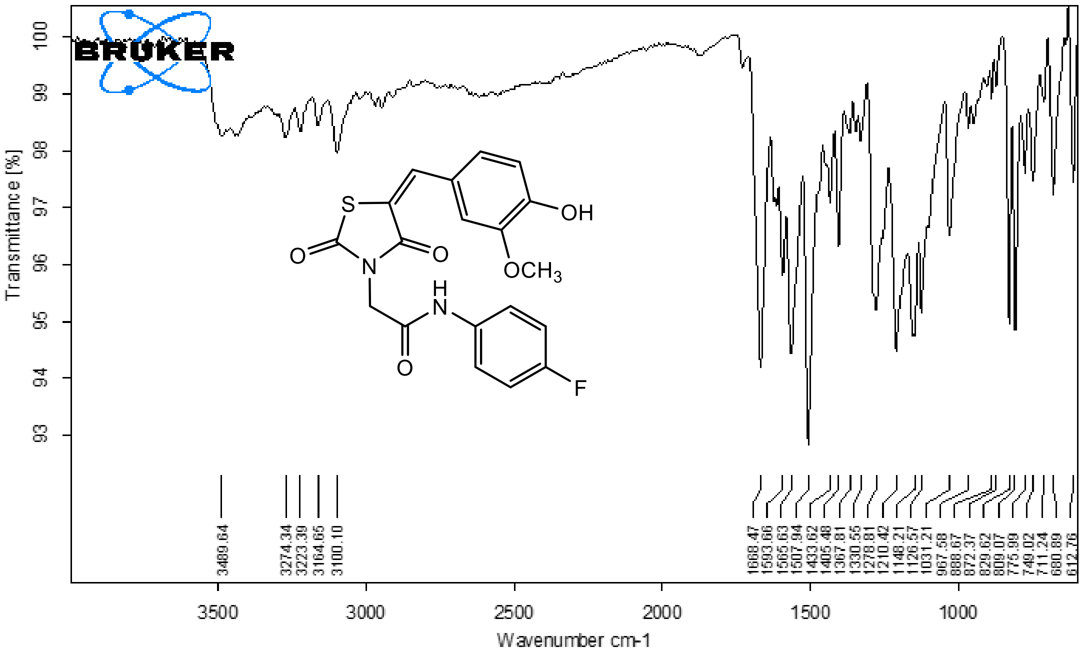


**Figure S17: IR spectrum of Compound D5: (E)-N-(4-fluorophenyl)-2-(5-(4-hydroxy-3-methoxybenzylidene)-2,4-dioxothiazolidin-3-yl)acetamide**

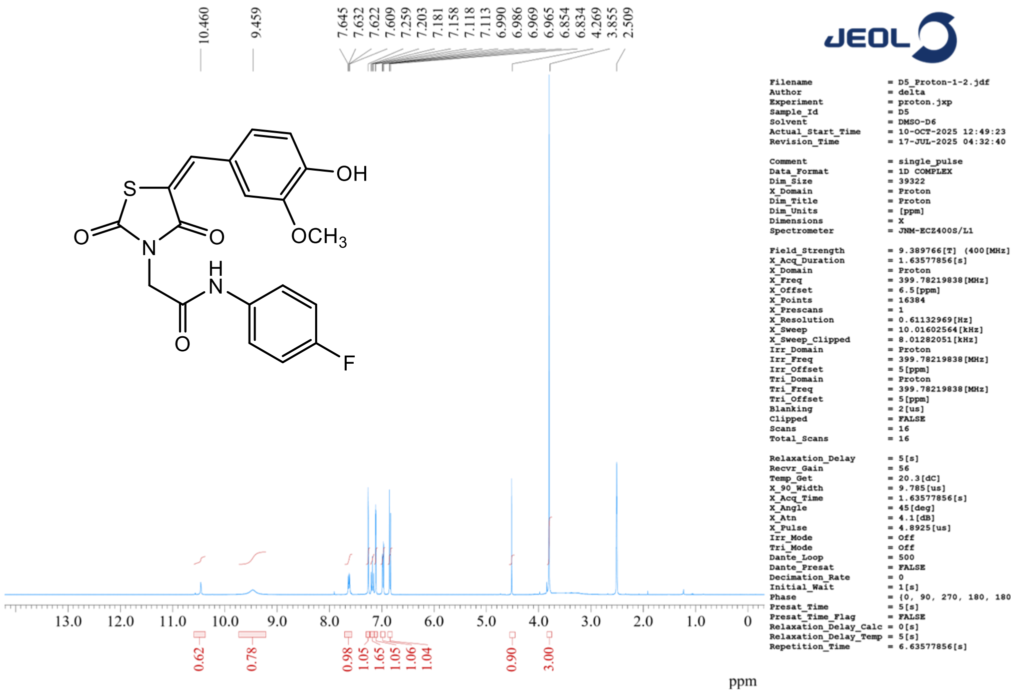


**Figure S18: 1H NMR spectrum of Compound D5: (E)-N-(4-fluorophenyl)-2-(5-(4-hydroxy-3-methoxybenzylidene)-2,4-dioxothiazolidin-3-yl)acetamide**

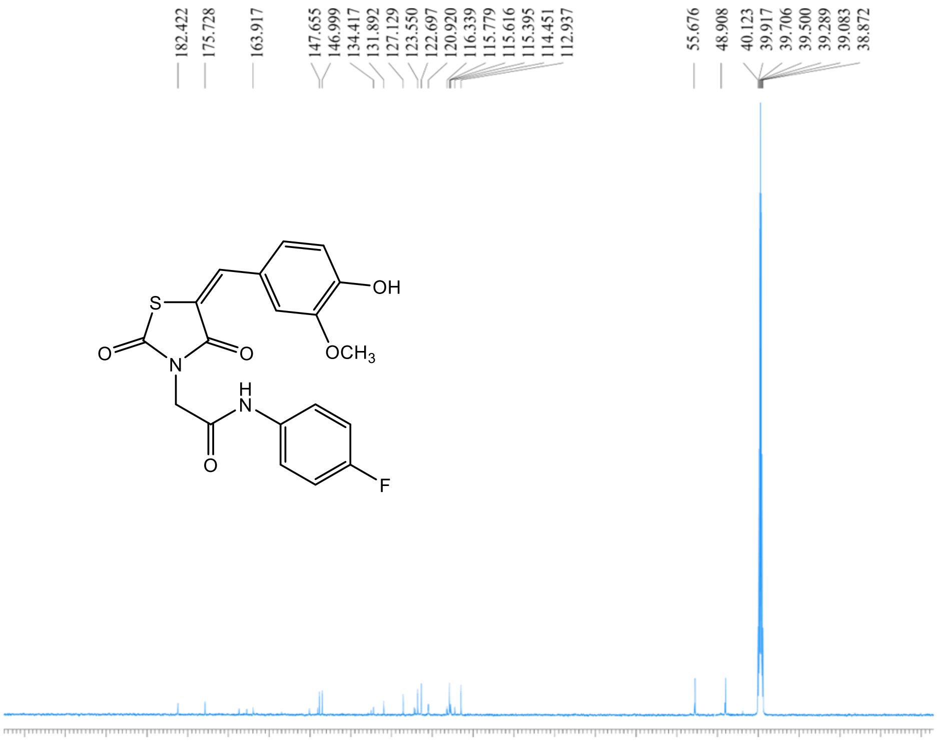


**Figure S19: 13C NMR spectrum of Compound D5: (E)-N-(4-fluorophenyl)-2-(5-(4-hydroxy-3-methoxybenzylidene)-2,4-dioxothiazolidin-3-yl)acetamide**

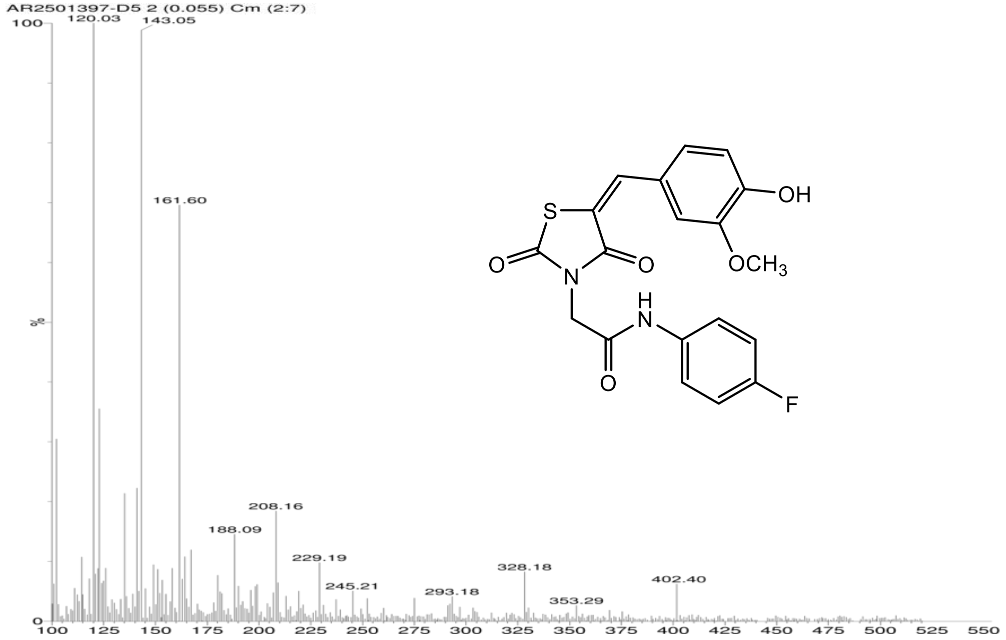


**Figure S20: Mass spectrum of Compound D5: (E)-N-(4-fluorophenyl)-2-(5-(4-hydroxy-3-methoxybenzylidene)-2,4-dioxothiazolidin-3-yl)acetamide**

| Code | SMILES |
|---|---|
| **D1** | O=C(NC1=CC=C(O)C=C1)CN(C/2=O)C(SC2=C\C3=CC=C(O)C(OC)=C3)=O |
| **D2** | O=C(NC1=CC=CC(O)=C1)CN(C/2=O)C(SC2=C\C3=CC=C(O)C(OC)=C3)=O |
| **D3** | O=C(NC1=CC=CC=C1O)CN(C/2=O)C(SC2=C\C3=CC=C(O)C(OC)=C3)=O |
| **D4** | O=C(NC1=CC=C([N+]([O])=O)C=C1)CN(C/2=O)C(SC2=C\C3=CC=C(O)C(OC)=C3)=O |
| **D5** | O=C(NC1=CC=C(F)C=C1)CN(C/2=O)C(SC2=C\C3=CC=C(O)C(OC)=C3)=O |
